# Erratum: Speckle-modulating optical coherence tomography in living mice and humans

**DOI:** 10.1038/ncomms16131

**Published:** 2017-07-11

**Authors:** Orly Liba, Matthew D. Lew, Elliott D. SoRelle, Rebecca Dutta, Debasish Sen, Darius M. Moshfeghi, Steven Chu, Adam de la Zerda

Nature Communications 8: Article number: 15845 ; DOI: 10.1038/ncomms15845 (2017); Published: 06 20 2017; Updated: 07 11 2017

An incorrect version of the Supplementary Information was inadvertently published with this Article which included incorrect figure references in Supplementary Table 4. The Article has now been updated to include the correct version of the Supplementary Information.

