# Supplementary material for: Speckle-modulating optical coherence tomography in living mice and humans
Source: Nat Commun. 2017 Jun 20;8:15845. doi: 10.1038/ncomms15845 (PMC5481831; doi:10.1038/ncomms15845)
Supplement: Supplementary Information [file ncomms15845-s1.pdf]

Type of file: PDF
Size of file: 0 KB
Title of file for HTML: Supplementary Information
Description: Supplementary Figures, Supplementary Tables, Supplementary Notes, Supplementary Discussion and Supplementary References.

Type of file: MOV
Size of file: 0 KB
Title of file for HTML: Supplementary Movie 1
Description: **The movie scans through a volume of a mouse pinna acquired with OCT (top) and SM-OCT (bottom).** The sampling spacing is 4 µm in both lateral directions. Sampling in the vertical dimension is 2 µm. After reconstruction, the images were resampled to obtain a voxel size of 2 µm in all three dimensions. Both scans were acquired using 20 B-scan averages. The full field of view is 2 x 2 mm, and was acquired by scanning and cascading four separate volumes of 2 x 0.5 mm (500 x 125 A-scans). The volume was cropped in the vertical direction and the intensity was scaled. In addition, we applied a gain on each A-scan to compensate for signal variations due to external causes.

Type of file: MOV
Size of file: 0 KB
Title of file for HTML: Supplementary Movie 2
Description: **The movie scans through a volume of a mouse pinna acquired with OCT (top) and SM-OCT (bottom).** The acquired volume is similar to that shown in Supplementary Movie 1, but shown as a volumetric three-dimensional rendering.

Type of file: MOV
Size of file: 0 KB
Title of file for HTML: Supplementary Movie 3
Description: **Segmentation of structures in the ear volume is enabled in SM-OCT owing to the removal of speckle noise.** SM-OCT reveals the structure of the epidermis, hair follicles and lymph vessels in three dimensions, which were highlighted in this movie by manual segmentation. The SM-OCT volume is similar to the one described in Supplementary Movie 1. Segments are color encoded as follows: cyan-epidermis, gray- auricular cartilage, magenta- lymph vessels, red- sebaceous glands, blue- follicle bulb, yellow- follicle shaft, green-unidentified part of the follicle.

Type of file: MOV
Size of file: 0 KB
Title of file for HTML: Supplementary Movie 4
Description: **The movie scans through a volume of a human fingertip acquired with OCT (top) and SM-OCT (bottom).** The sampling spacing is 4 µm in both lateral directions. Sampling in the vertical dimension is 2 µm. After reconstruction, the images were resampled to obtain a voxel size of 4 µm in all three dimensions. Both scans were acquired using 20 B-scan averages. The full field of view is 3.2 x 0.5 mm. The volume was cropped in the vertical direction and the intensity was scaled.

Type of file: PDF
Size of file: 0 KB
Title of file for HTML: Peer Review File
Description:

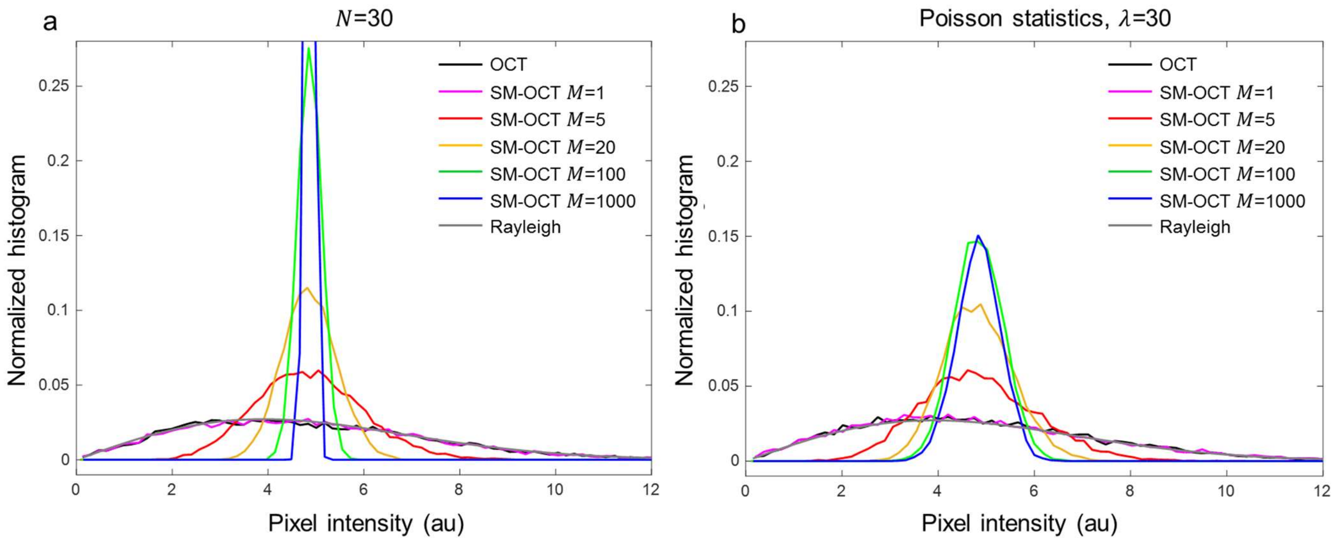

Supplementary Figure 1| Normalized histograms of the pixel values obtained with a Monte-Carlo simulation of Supplementary Eq. 1 with 1, 5, 20, 100 and 1000 averages. (**a**) The results of the simulation with 30 particles in each voxel. (**b**) The results of the simulation with the number of particles in each voxel randomly chosen from a Poisson distribution with λ=30. Due to the random distribution of particles, the variance of pixel values does not reduce to zero. This non-zero variance explains why the images of phantoms composed of particles dispersed in a volume do not appear completely uniform with SM-OCT even with 100 averages.

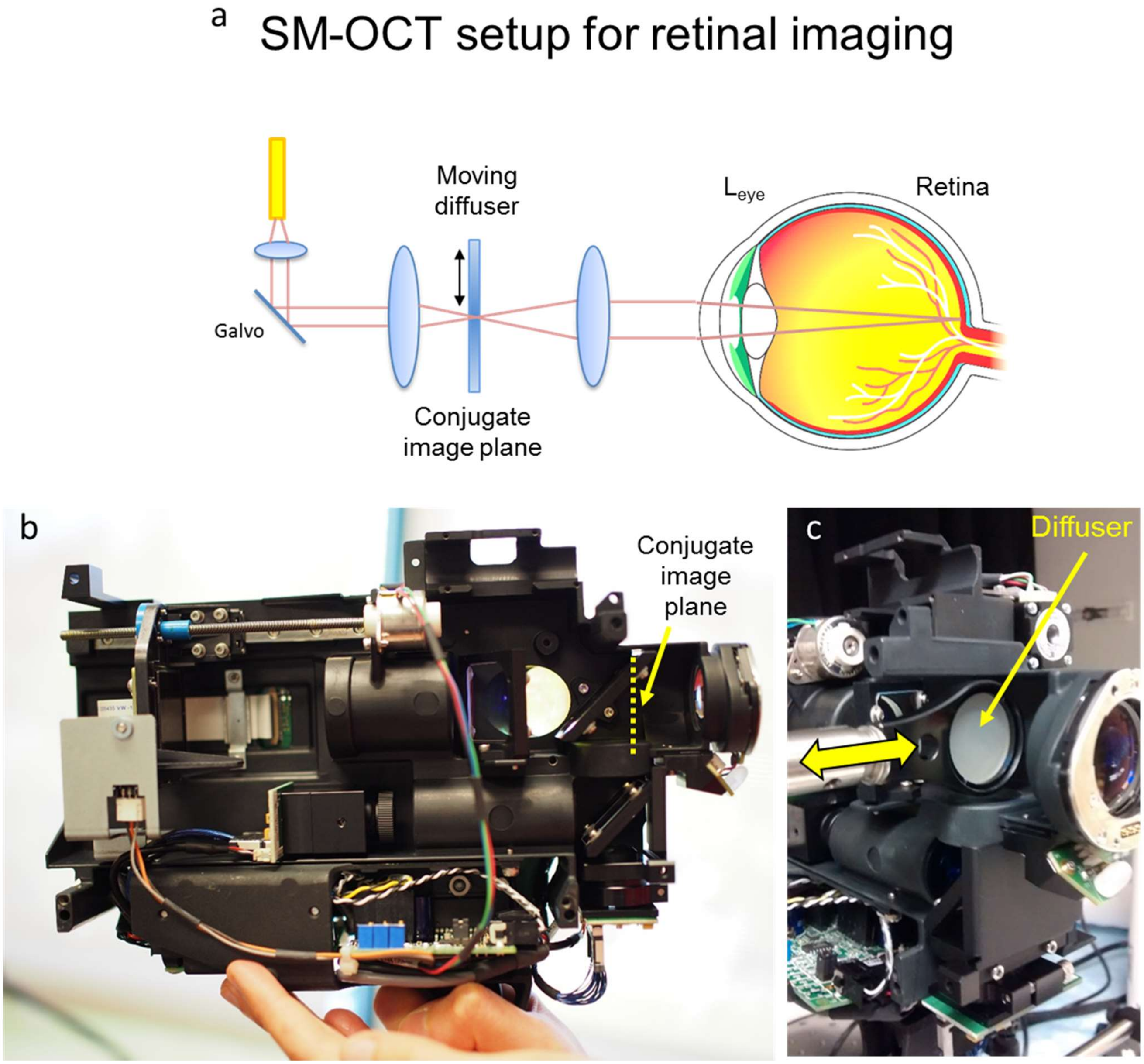

Supplementary Figure 2| Schematic and implementation of SM-OCT for a human ophthalmic imaging system. (**a**) A schematic showing the placement of the moving diffuser in the optical path of an ophthalmic OCT. (**b**) The interior of the scan-head of the iFusion (Optovue). The conjugate image plane is marked by the dashed yellow line. (**c**) The scan-head with the diffuser placed in the conjugate image plane. The yellow arrow shows the direction of the motion of the diffuser.

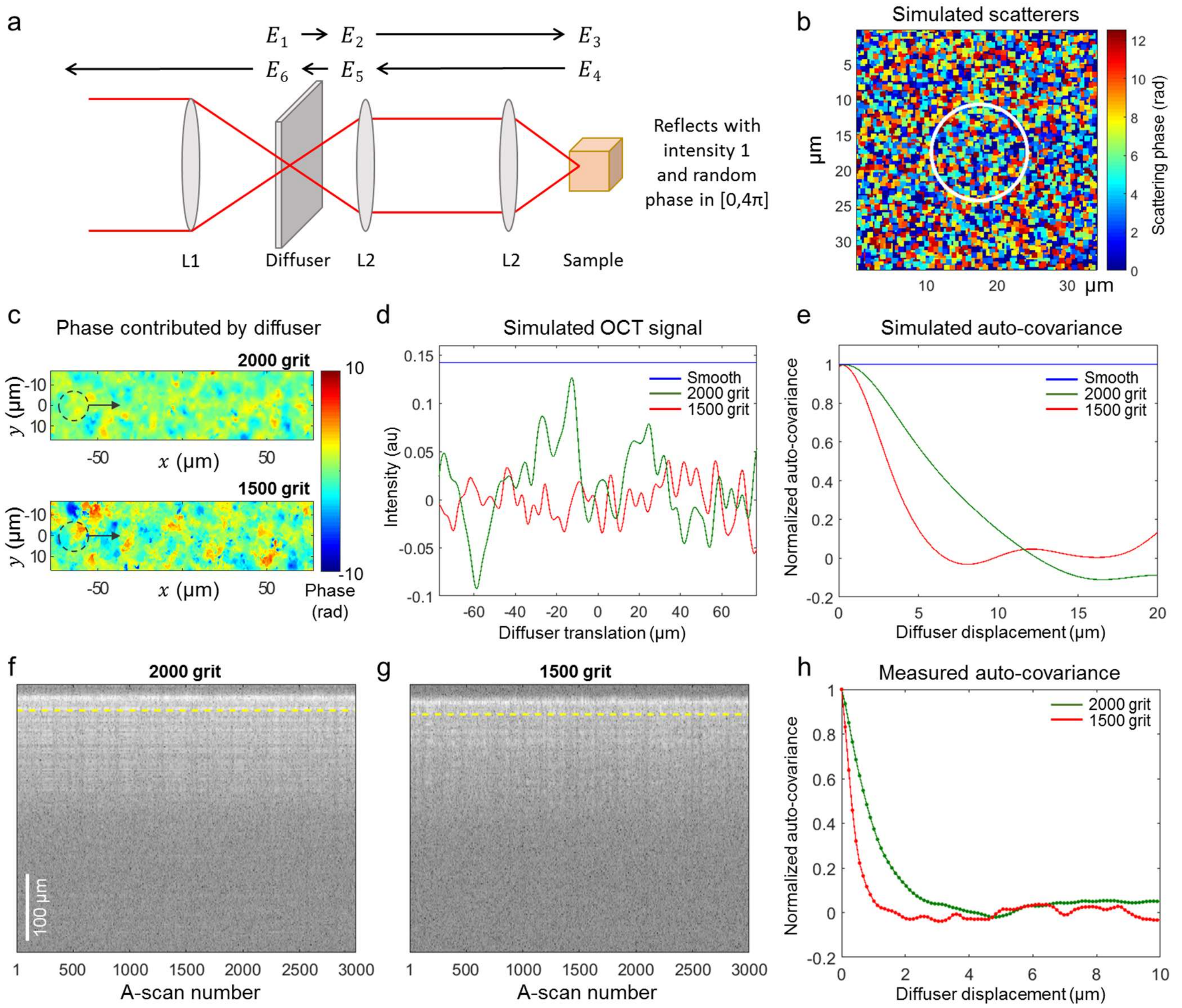

Supplementary Figure 3| Simulation and measurement of speckle decorrelation in SM-OCT. (**a**) Schematic of light propagation through the SM-OCT sample arm. (**b**) A simulated sample, composed of 4,000 scatterers with size 0.8x0.8 μm^2 at random locations that reflect the incident electric field with an amplitude ratio of 1 and a random phase in the interval [0, 4π]. The white circle represents the $1/e^2$ width of the input OCT Gaussian beam. (**c**) The phase contribution to the propagating OCT beam by the 2000 grit (top) and 1500 grit (bottom) diffusers. The phase contribution is calculated using the depth profile measurements of the diffusers used in this study. The dashed circle represents the $1/e^2$ width of the incident OCT Gaussian beam and the arrow shows the direction of its displacement relative to the surface of the diffuser. (**d**) The OCT signal obtained from a simulated optical setup with a smooth diffuser, 2000 grit diffuser, and 1500 grit diffuser as a function of the translation of the diffuser. (**e**) The normalized auto-covariance of the simulated OCT signals in (**d**). The simulation predicts faster changes in the OCT signal when using the rougher diffuser (1500 grit). (**f,g**) A-scans of a single location on a TiO_2+PDMS phantom, with the diffusers moving at 2.25 mm s^{-1}. (**h**) The normalized auto-covariance of the OCT signal inside the phantom, calculated at a depth shown by the dashed lines in (**f,g**). As predicted by the simulation (**e**), signal change (speckle variation) is achieved with a smaller diffuser translation when using the rougher diffuser (1500 grit) compared to the finer diffuser (2000 grit).

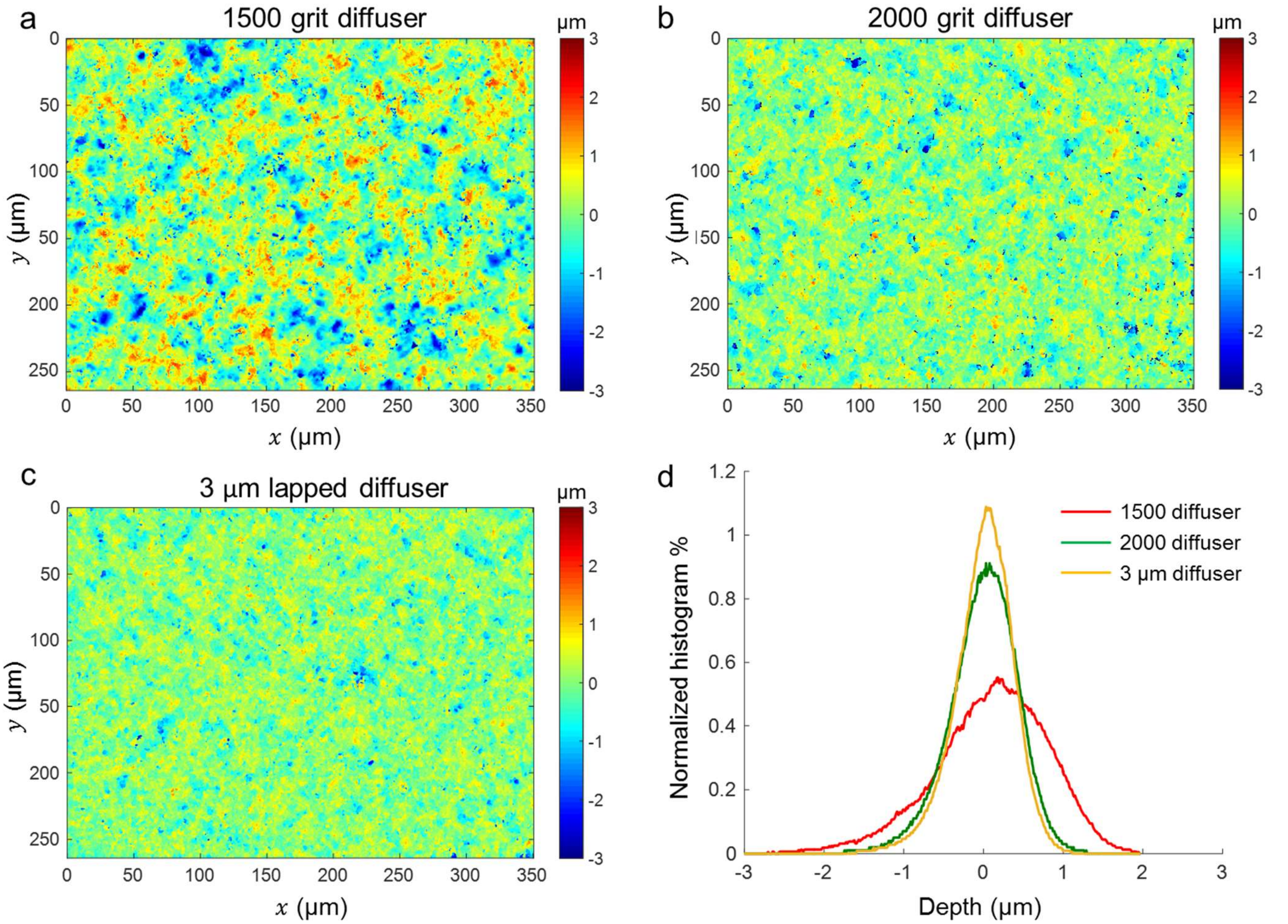

Supplementary Figure 4| Characterization of the thickness profile of the diffusers. (**a**) Depth profile of the 1500 grit diffuser. (**b**) Depth profile of the 2000 grit diffuser. (**c**) Depth profile of the 3 μm lapped diffuser. (**d**) Depth histogram of all of the diffusers used in this study.

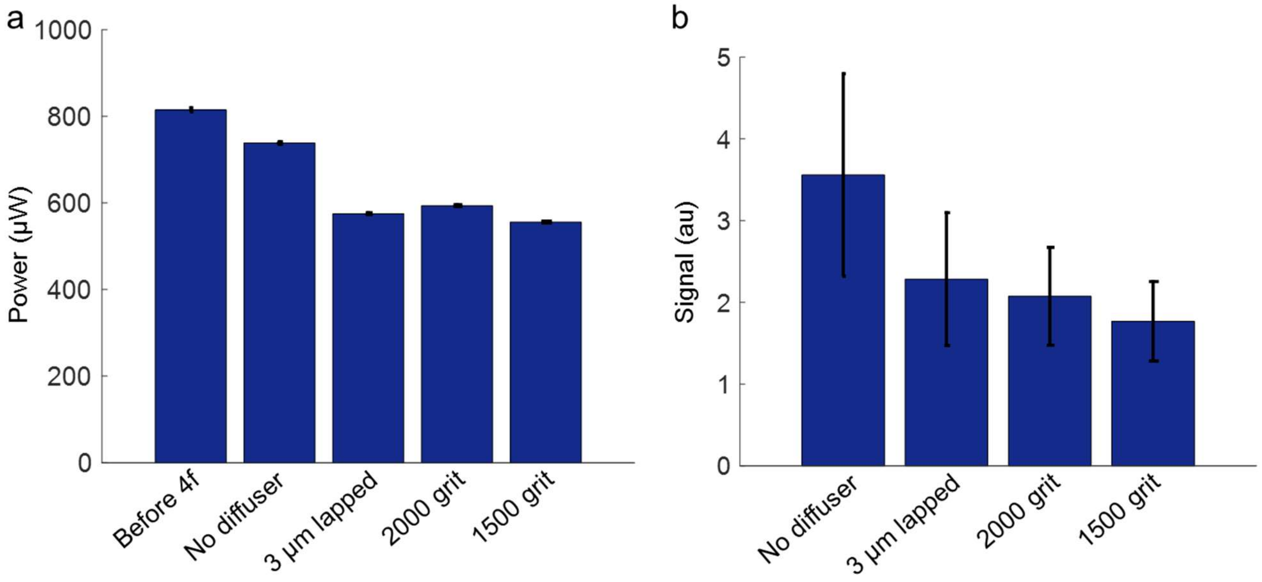

Supplementary Figure 5| Power and signal intensity of OCT and SM-OCT with the three diffusers. (**a**) Incident power on the sample for OCT and SM-OCT, based on Supplementary Table 1. (**b**) Signal intensity of OCT and SM-OCT, based on Supplementary Table 2. Error bars are the standard deviation of the measurements.

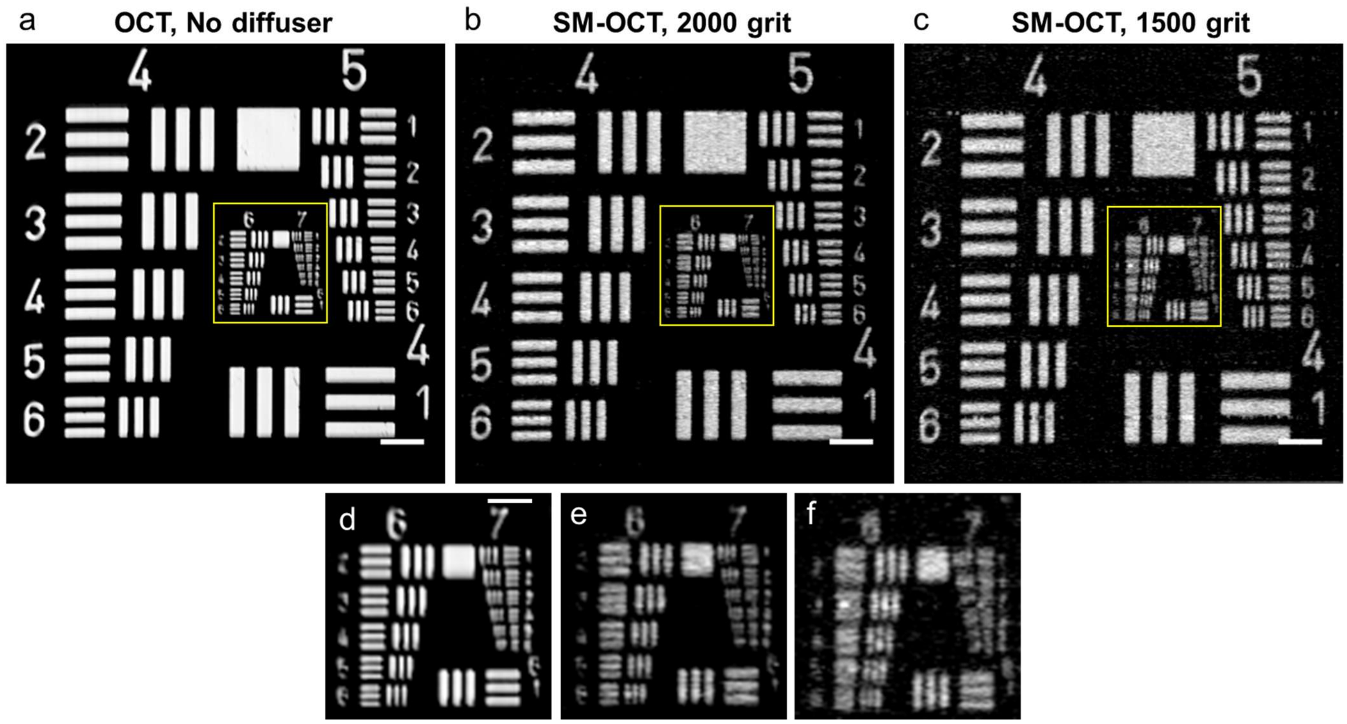

Supplementary Figure 6| Lateral resolution of OCT and SM-OCT with two diffusers, measured on a 1951 USAF resolution test target. (**a,d**) OCT, (**b,e**) SM-OCT with 2000 grit diffuser, and (**c**,**f**) SM-OCT with 1500 grit diffuser. Scale bars in panels (**a**), (**b**) and (**c**) are 100 µm. Scale bar on panel (**d**), which also applies to (**e**) and (**f**), is 50 µm.

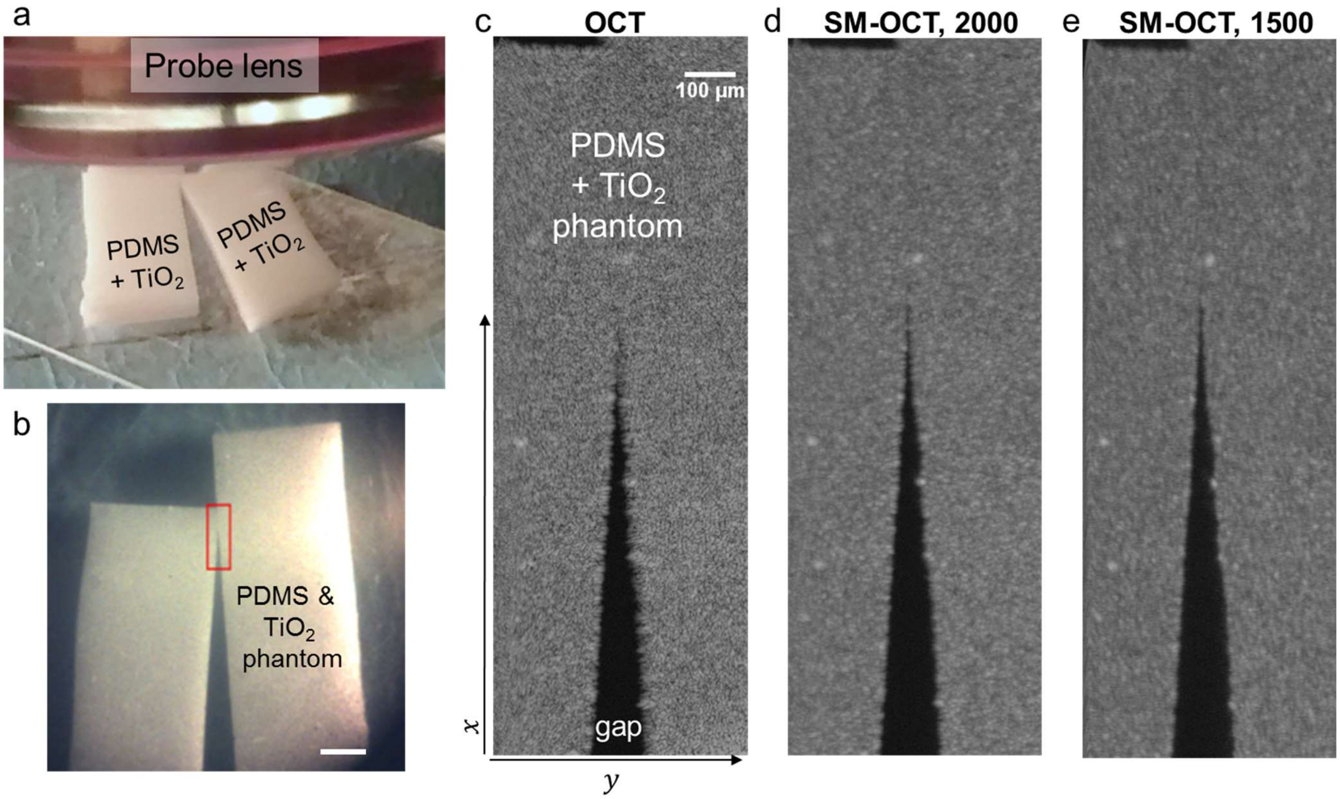

Supplementary Figure 7| Visibility of closely-spaced scattering objects with OCT and SM-OCT. A phantom composed of PDMS and TiO_2 powder was shaped to form a gap of decreasing size to evaluate the effective spatial resolution of SM-OCT versus OCT. (**a**) A view of the phantom beneath the OCT probe, showing the two parts of PDMS and the gap between them. (**b**) The phantom as imaged by the camera inside the OCT probe. The red rectangle shows the location of the scanned volume. Scale bar is 1 mm. (**c**) OCT *en face* image inside of the phantom. (**d**) Similar scan using SM-OCT with 2000 grit diffuser. (**e**) Similar scan using SM-OCT with 1500 grit diffuser.

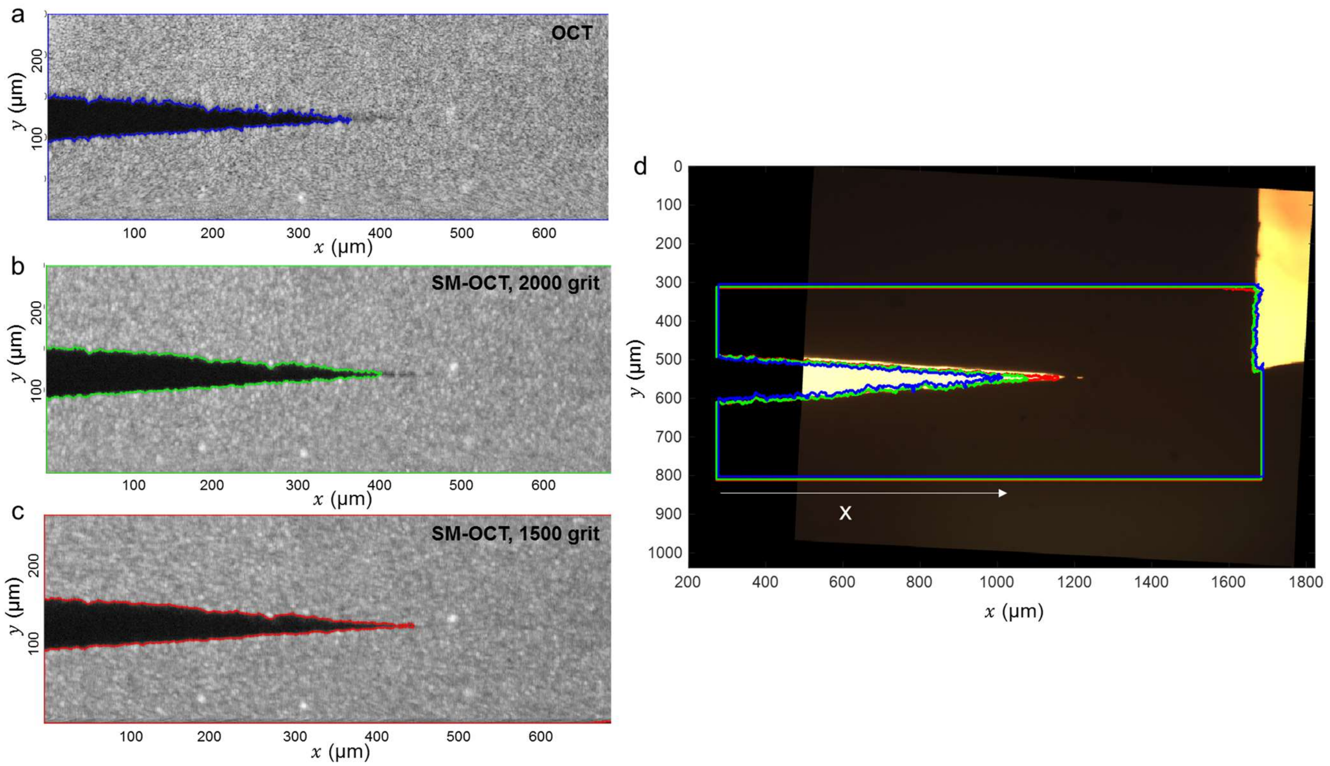

Supplementary Figure 8| Segmentation and registration of the images. (**a-c**) OCT and SM-OCT scans annotated with lines that represent the segmentation boundary between the phantom and the gap. The images were segmented with an intensity-based threshold, as detailed in Supplementary Note 3. (**d**) The segmentation boundaries drawn on top of a registered image of the phantom obtained with a bright-field microscope (10x, NA=0.25).

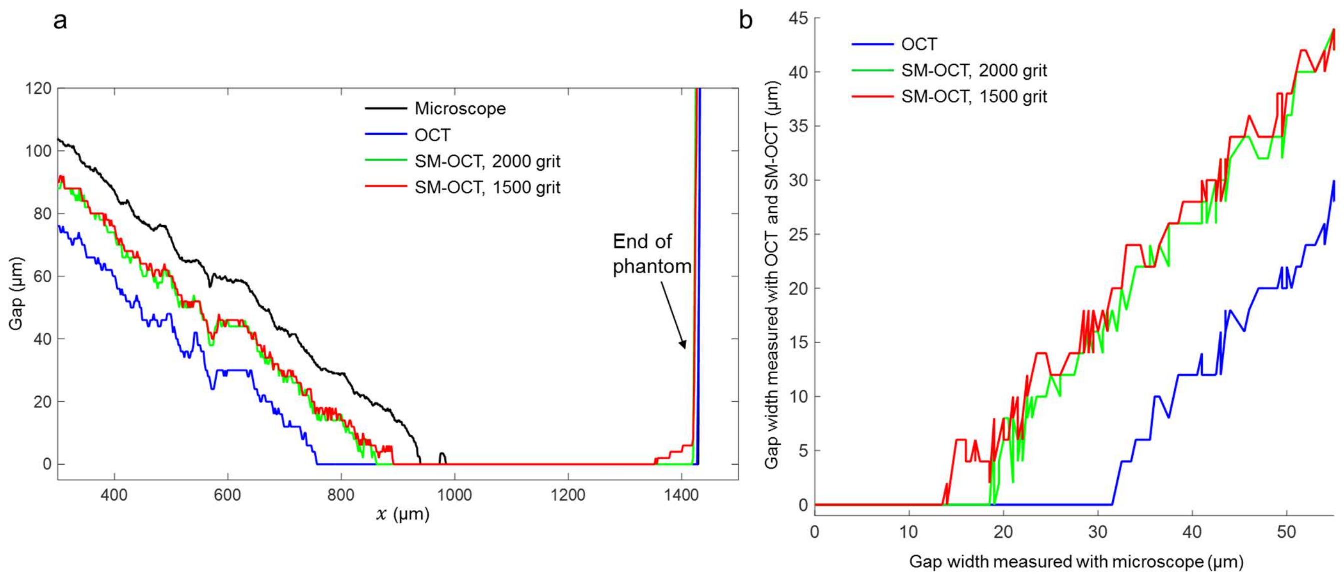

Supplementary Figure 9| The width of the gap in the turbid phantom, measured by OCT, SM-OCT and a bright field microscope. (**a**) The size of the gap as a function of location (x) as measured from Supplementary Fig. 8d. (**b**) The size of the gap in the OCT and SM-OCT images, as shown in (**a**), plotted as a function of the size of the gap in the microscope image. The difference in visibility between OCT and SM-OCT is clearly shown. The effective resolution in SM-OCT is 2.5-fold better than OCT.

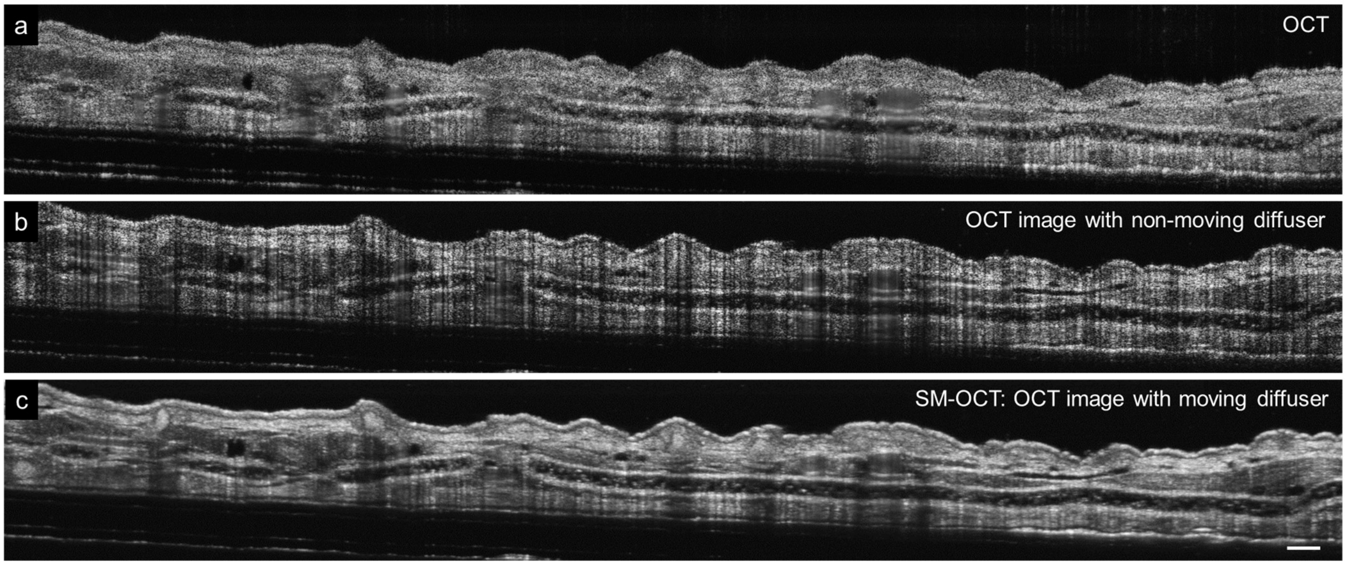

Supplementary Figure 10| The effect of the diffuser on the OCT image and signal intensity. (**a**) OCT image of a mouse pinna. (**b**) OCT image of the same location as (**a**) with a static diffuser. The vertical lines depict local intensity changes due to the pattern of the diffuser. (**c**) OCT image with a moving diffuser, generating the SM-OCT effect. All three images are composed of 100 intensity averaged B-scans. Scale bar is 100 μm.

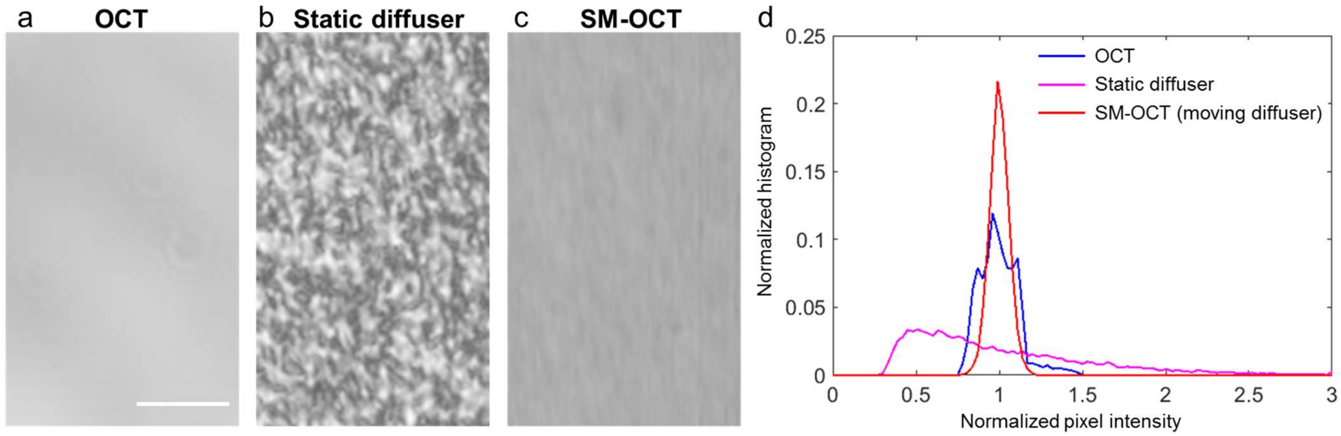

Supplementary Figure 11| Characterization of the illumination variability originating from the diffuser. *en face* images of a glass slide (averaged over the depth and 100 frames, shown in a similar logarithmic scale) scanned with (**a**) no diffuser, (**b**) a static diffuser and (**c**) a moving diffuser. Scale bar is 100 μm. The image with the static diffuser (**b**) shows the variability in the illumination of the sample due to the diffuser. The SM-OCT image (**c**), which was acquired with a moving diffuser, shows the reduction in illumination variability obtained with averaging. (**d**) Normalized histograms of the normalized pixel intensity (the signal of each pixel divided by average signal in the image, in linear scale) for the images shown in (**a-c**). The histograms show that the illumination statistics change from a Rayleigh distribution to a much narrower distribution following averaging over 100 frames, indicating that on average the illumination-uniformity of the sample is improved. Furthermore, the diffuser was able to remove diffraction artifacts that broadened the intensity distribution in the OCT image. Therefore, overall, SM-OCT was able to improve the illumination-uniformity compared to OCT. The standard deviation of the normalized pixel intensities is 0.12, 0.53 and 0.06 for OCT, static diffuser, and SM-OCT, respectively.

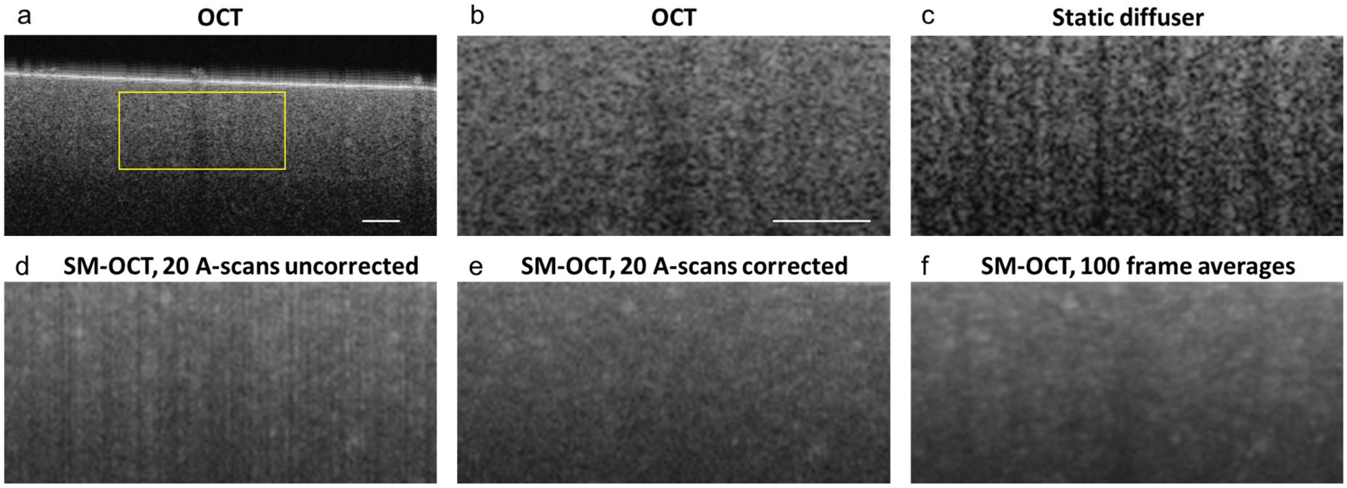

Supplementary Figure 12| Residual illumination variability and its reduction in post-processing. (**a**) OCT B-scan of a phantom composed of PDMS and TiO_2 powder. (**b**) A close-up view on the region shown in (**a**). (**c**) The same region, acquired with a static diffuser. (**d**) SM-OCT image with 20 A-scan averages. Speckle noise is reduced; however, the image shows vertical line artifacts due to illumination variability that is not entirely removed by averaging. (**e**) The image in (**d**), after correction of the vertical-line artifact in post-processing. (**f**) SM-OCT image with 100 B-scan (frame) averages and a moving diffuser. The vertical line artifacts are not visible and speckle noise is significantly reduced, revealing the variable distribution of TiO_2 in the phantom.

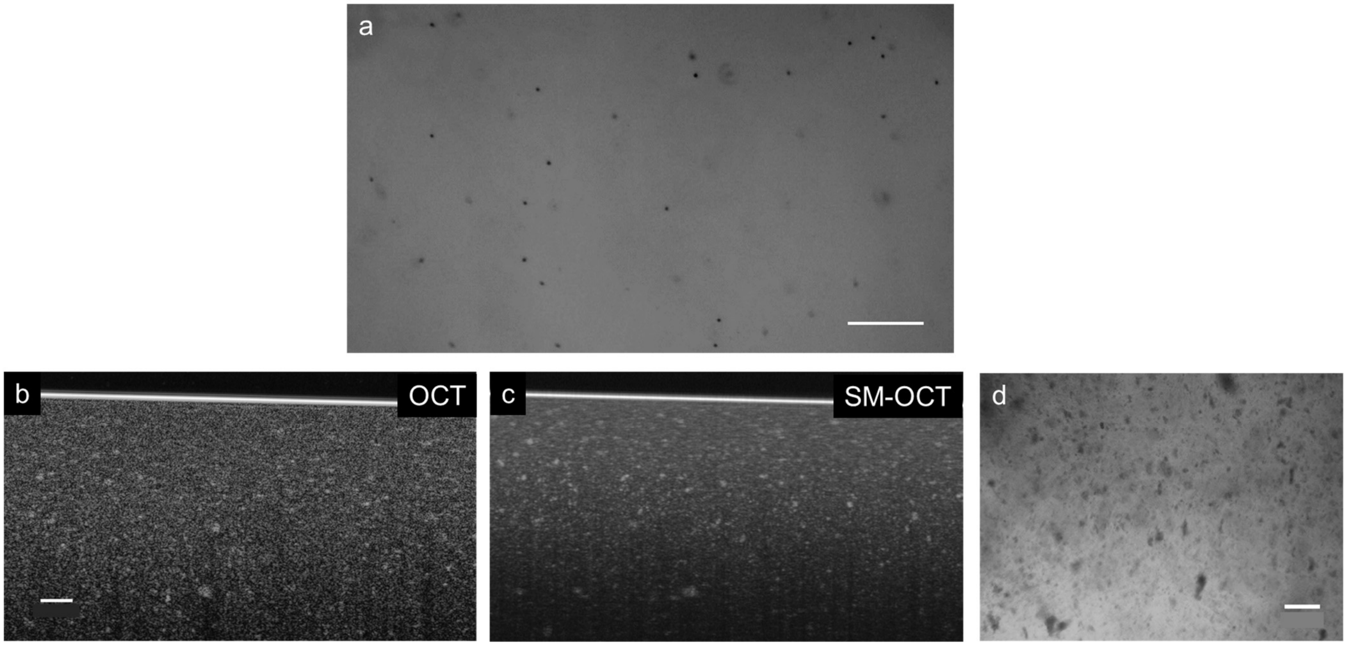

Supplementary Figure 13| Validation of the capability of SM-OCT to produce images that better represent the true structure of the sample. (**a**) Intensity image (gray-scale) from a bright field microscope of a thin slice of an agarose-LGNR phantom with 3 μm diameter beads (10x, NA = 0.25). The image shows the beads sparsely dispersed in a uniform phantom. The LGNRs are too small to be resolved individually. Scale bar is 100 μm. (**b,c**) OCT and SM-OCT cross-sectional scans of an agarose phantom with TiO_2 nanopowder. The nanopowder does not disperse well in the phantom; rather, it forms large clumps. These clumps are hidden within the speckle noise in the OCT image, but are revealed in the SM-OCT image. Scale bar is 100 μm. (**d**) A bright field microscope (gray-scale) image of the phantom presented in (**b,c**) showing the shape size and distribution of the clumps in the agarose base. The comparison of SM-OCT images to microscope images shows that SM-OCT provides a close representation of the true structure of the phantoms. Scale bar is 50 μm.

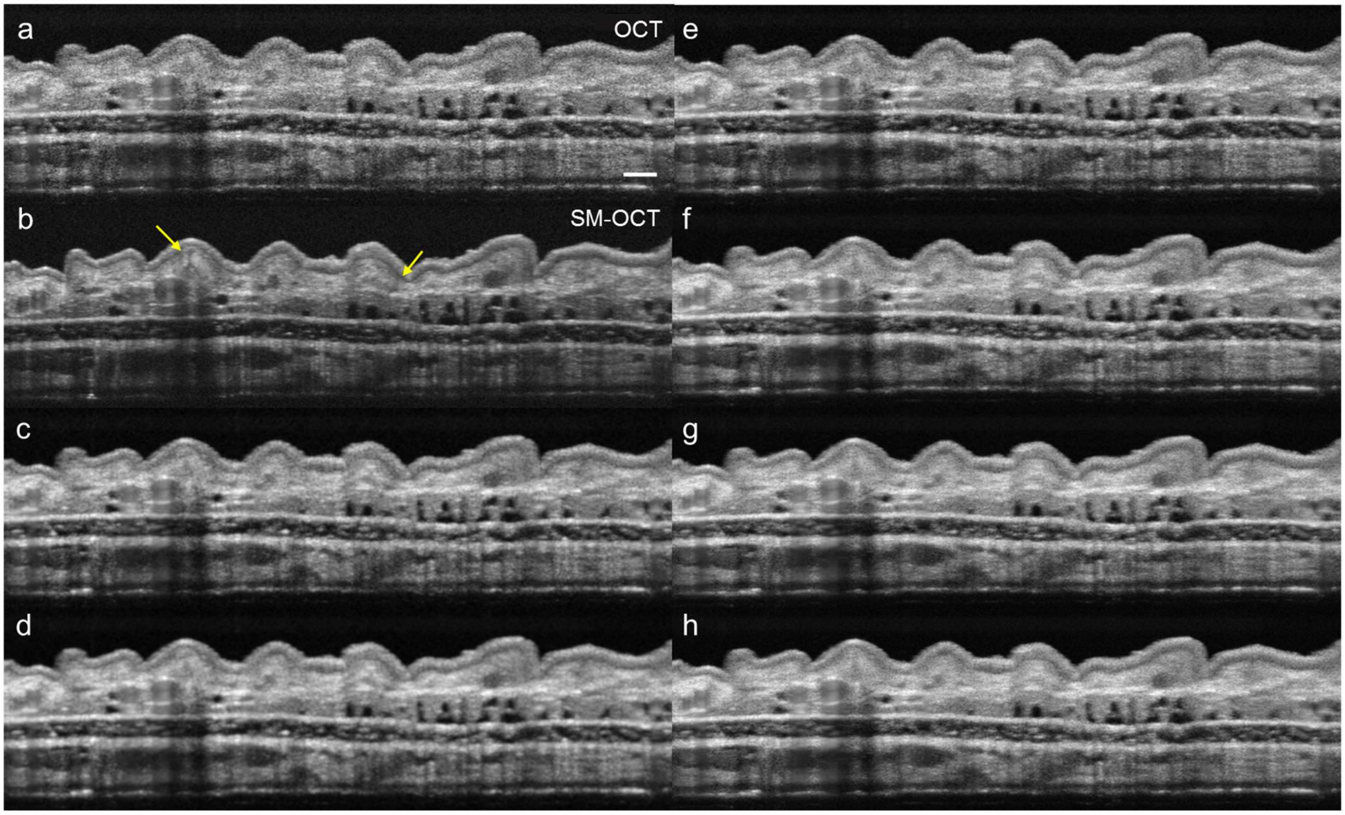

Supplementary Figure 14| Comparison of SM-OCT to spatial compounding and 3D smoothing. (**a**) OCT scan sampled every 4 µm in both lateral directions, with 20 B-scan averages. The image was created by averaging 2 adjacent frames (to improve signal-to-noise, averaging is of linear-scale images) and later resampled to obtain a voxel size of 2 µm in all three directions. Averaging 2 scans, which span 4 µm, does not reduce speckle because the averaged scans are inside the PSF. Scale bar is 100 µm. (**b**) SM-OCT scan, with the 1500 grit diffuser, using the same acquisition parameters and post processing as (**a**). (**c,d**) Images resulting from three-dimensional smoothing of the OCT volume described in (**a**). Smoothing is done on the linear-scale image after resampling using Matlab's smooth3 function with a Gaussian kernel in a square window with a size of 11 pixels. The standard deviation of the Gaussian is 0.95 in (**c**) and 1.25 in (**d**). (**e-h**) Averaging and processing of the OCT volume as described in (**a**), of 4, 7, 9, and 13 frames, respectively (spanning 12, 24, 32, and 48 µm). These images show that although spatial compounding reduces speckle noise, it does not reveal structure (yellow arrows) as well as SM-OCT.

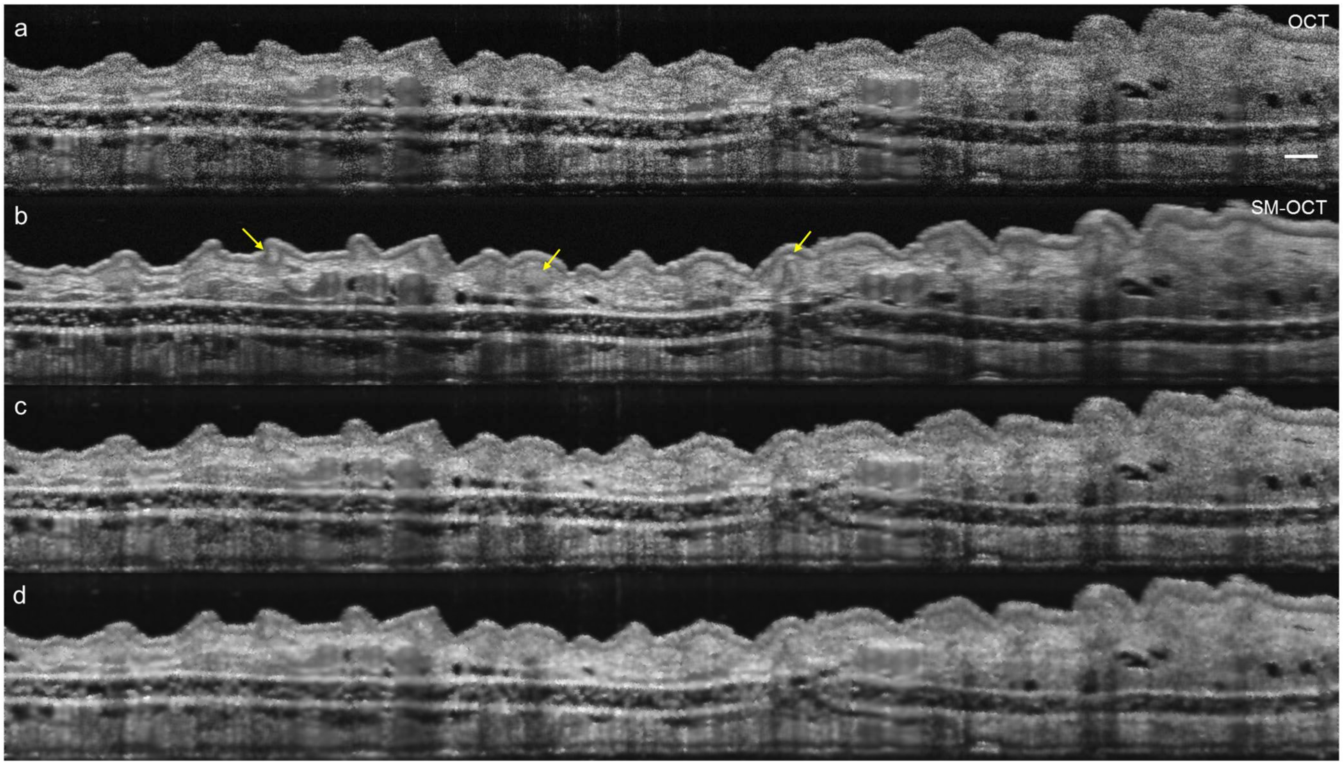

Supplementary Figure 15| Comparison of SM-OCT to 2D digital filtering methods for speckle removal. (**a**) OCT image of a mouse pinna. The pixel size is 2x2 μm. Scale bar is 100 μm. (**b**) SM-OCT image at a similar location. Yellow arrows point to various fine structures. (**c,d**) The OCT image post-processed with an adaptive Wiener filter using neighborhoods of sizes 5x5 and 7x7 pixels, respectively.

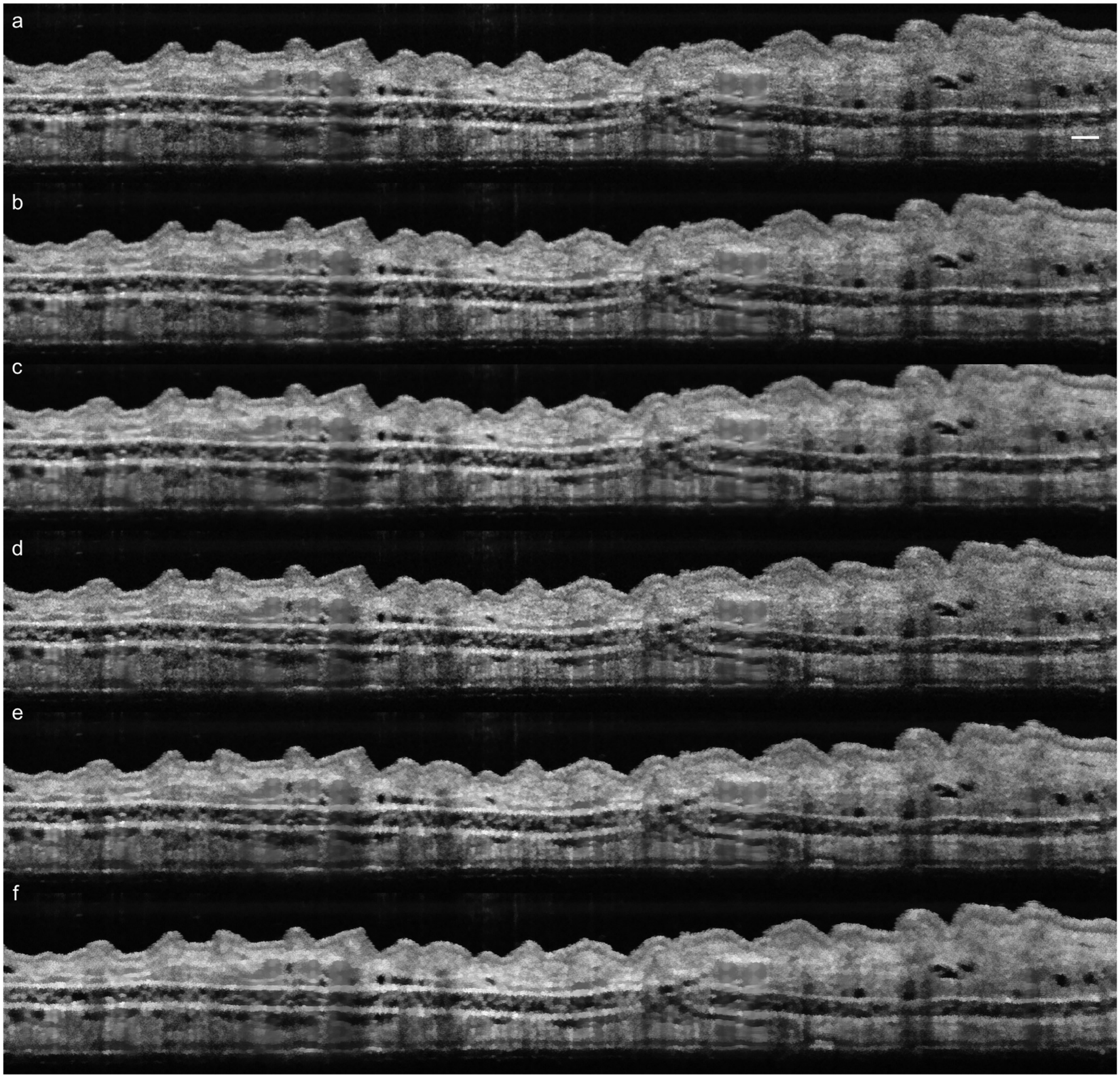

Supplementary Figure 16| Comparison of SM-OCT to 2D digital filtering methods for speckle removal (continued). (**a-c**) The OCT image post-processed with a hybrid median filter (HMF) using neighborhoods of sizes 5x5, 7x7, and 9x9 pixels, respectively. Scale bar is 100 μm. (**d-f**) The OCT image post-processed with a symmetric nearest-neighbor filter (SNN) using neighborhoods of sizes 5x5, 7x7, and 9x9 pixels, respectively. These examples show that filters of a small size do not reduce speckle noise sufficiently, whereas, larger filters create artifacts and compromise some of the information in the image.

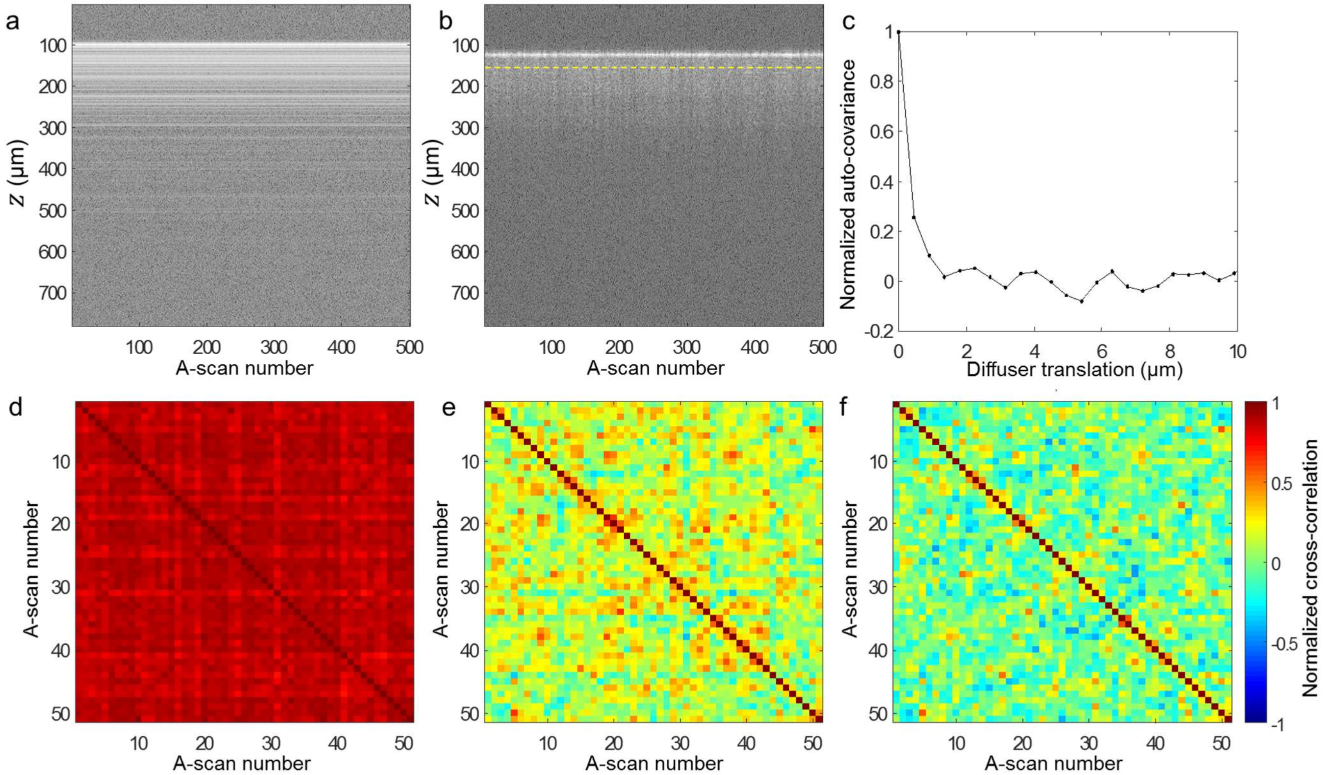

Supplementary Figure 17| Analysis of speckle decorrelation in SM-OCT with A-scan averaging. (**a**) 500 A-scans of a single location on a phantom, with diffuser (1500 grit) not moving. (**b**) 500 A-scans of a single location on a phantom, with the diffuser moving at 9 mm s^{-1}. (**c**) Normalized auto-covariance of the OCT signal inside the phantom, calculated at the depth shown by the dashed line in (**b**), as a function of the diffuser translation. (**d**) Normalized cross-covariance of the scan with the static diffuser, calculated on pixels inside of the phantom. (**e**) Similar to (**d**), calculated from the scan with the rotating diffuser. (**f**) Similar to (**e**), after subtracting the structure of the sample by subtracting the average across A-scans.

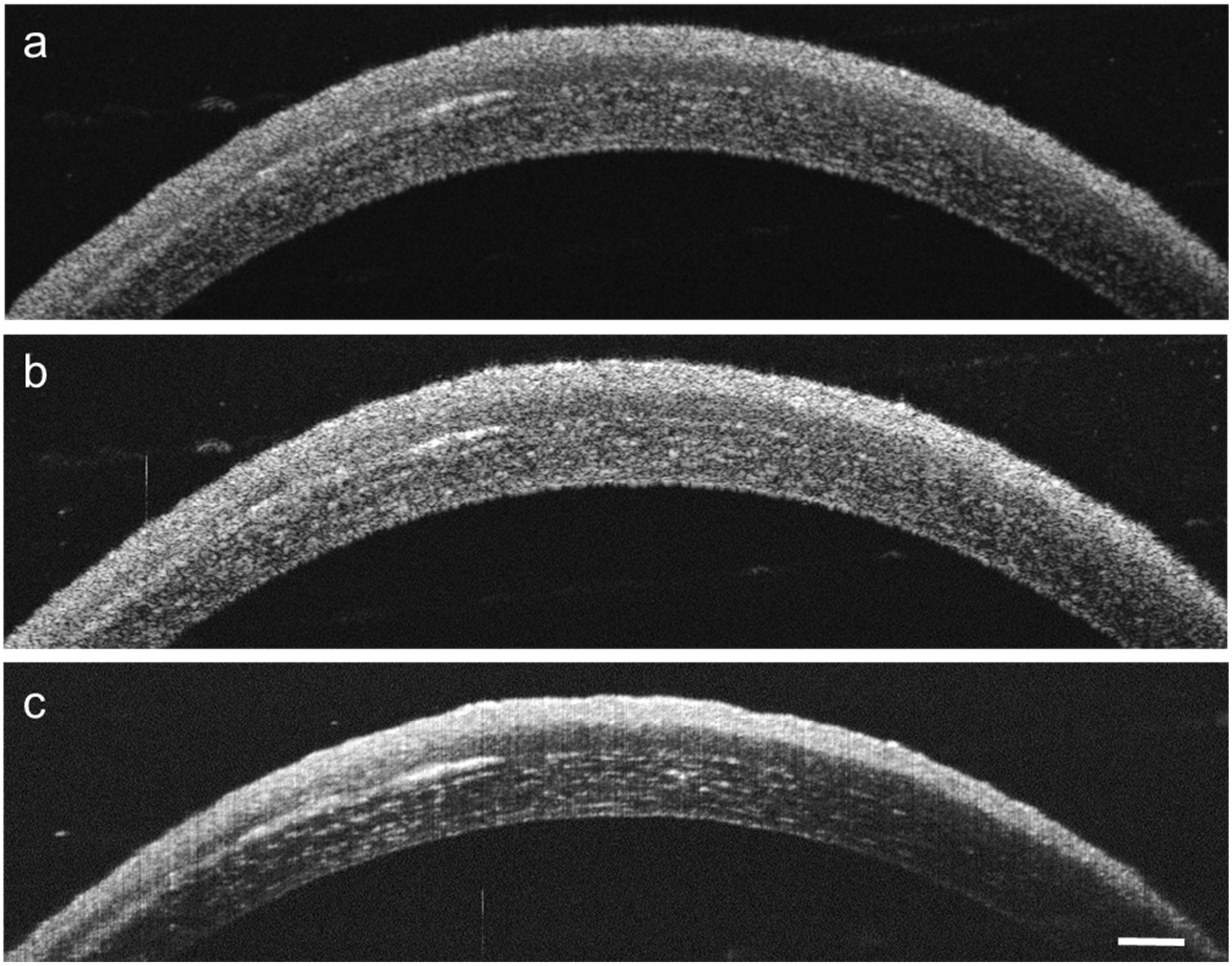

Supplementary Figure 18| OCT and SM-OCT images of a mouse cornea with A-scan averaging instead of frame averaging. (**a**) OCT image composed by averaging 100 frames (B-scans). (**b**) OCT image composed by averaging 100 consecutive A-scans. (**c**) SM-OCT image composed by averaging 100 consecutive A-scans. The speckle pattern is uncorrelated between A-scans owing to a fast moving (rotating) diffuser. The SM-OCT image shows the lamellar structure of the cornea, which is hidden by speckle noise in the OCT images. The left side of the cornea shows increased light scattering of unknown origin. Scale bar is 100 μm.

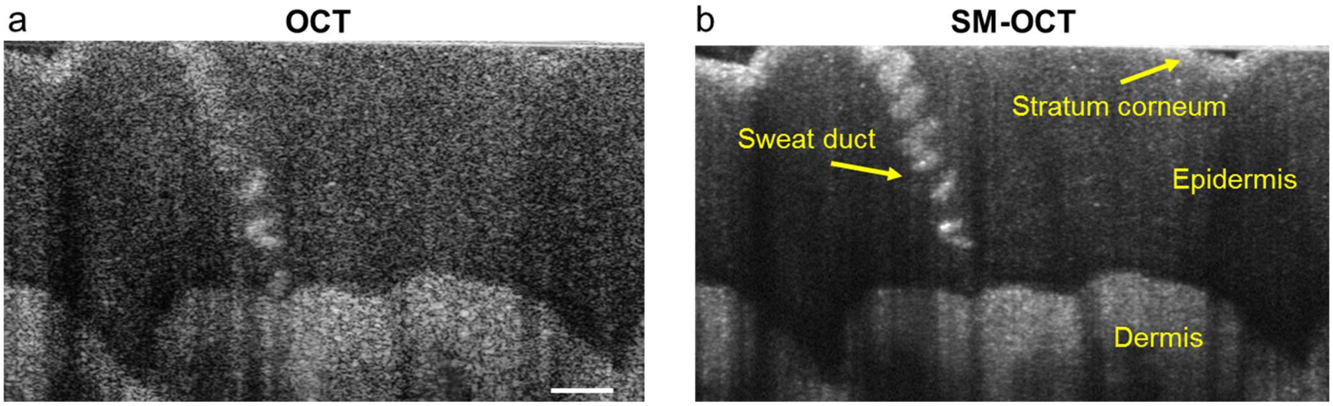

Supplementary Figure 19| OCT and SM-OCT images of intact human fingertip skin. (**a**) OCT B-scan of a fingertip showing a sweat duct. Scale bar is 100 μm. (**b**) SM-OCT B-scan of a similar region, showing the sweat duct in greater detail, along with a better view of the layers in the skin.

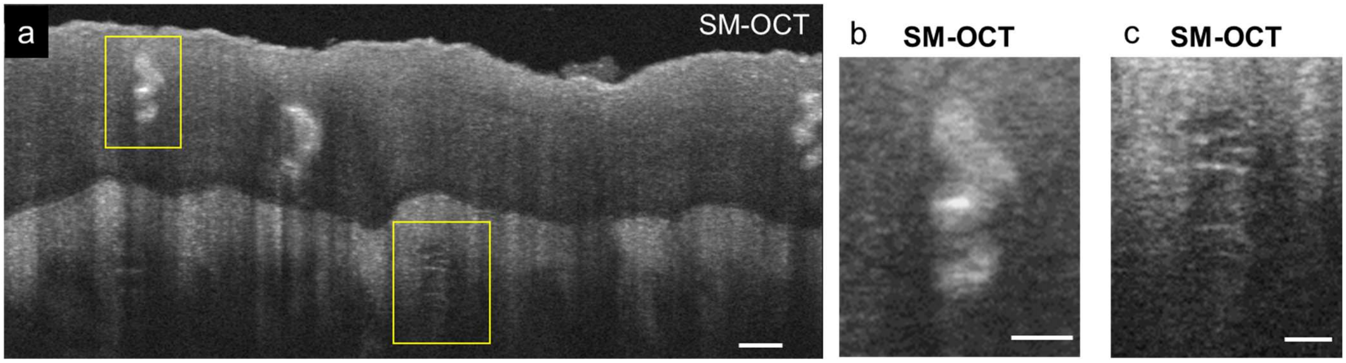

Supplementary Figure 20| SM-OCT image of intact human fingertip skin. The acquisition parameters are similar to the OCT image shown in Fig. 7 (**a**) SM-OCT B-scan of a fingertip. Scale bar is 100 μm. (**b**) Close-up view on the sweat duct marked in (**a**). Scale bar is 50 μm. (**c**) Close-up view on the tactile corpuscle marked in (**a**). Scale bars is 50 μm.

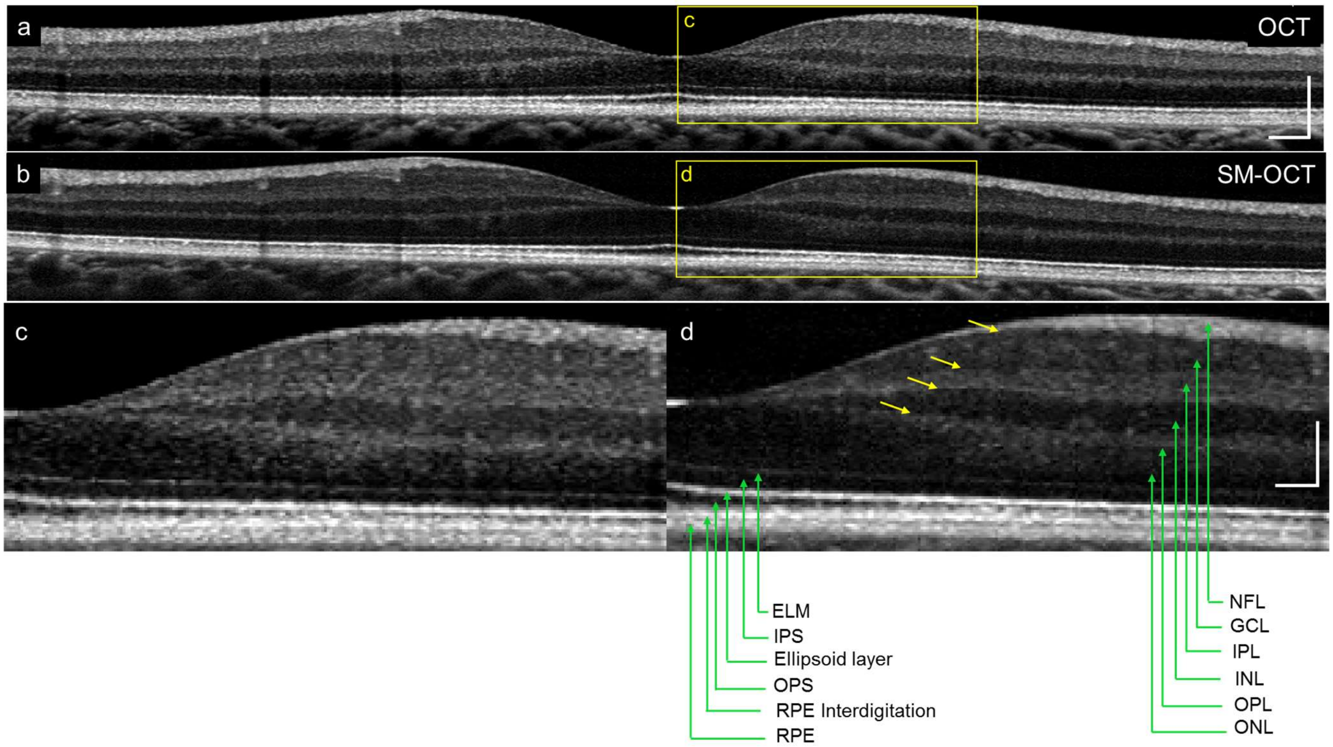

Supplementary Figure 21| SM-OCT imaging of human retina shows clearer differentiation between the retinal layers without loss of detail. (**a**) In the top panel, the normal human retina is seen through a B-scan (cross-section) of the fovea using commercially-available spectral domain OCT (SDOCT). Scale bar is 200 μm. (**b**) The same scan through the same human retina is shown using SM-OCT (middle panel). (**c**) Bottom left panel depicts magnification of the yellow box in (**a**) with SDOCT. (**d**) Bottom right panel depicts the corresponding region magnified in (**b**) using SM-OCT. Optical removal of speckle results in a clearer delineation of the various retinal layers, seen most clearly in the interfaces between the nuclear layers and the adjacent plexiform layers and/or nerve fiber layers (yellow arrows), the ellipsoid layer, OPS, RPE Interdigitation, and RPE. ELM=external limiting membrane, IPS=Inner photoreceptor segments, OPS=Outer photoreceptor segments, RPE=retinal pigment epithelium, NFL=nerve fiber layer, GCL=ganglion cell layer, IPL=inner plexiform layer, INL=inner nuclear layer, OPL=outer plexiform layer, ONL=outer nuclear layer. Scale bar is 100 μm.

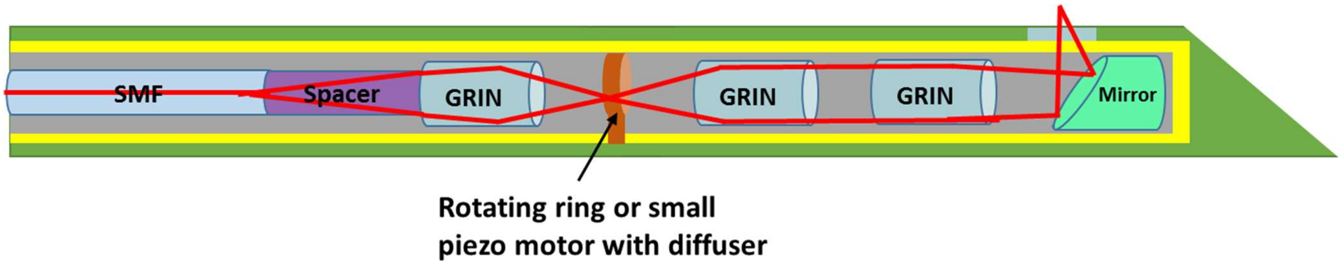

Supplementary Figure 22| Proposed design of an SM-OCT endoscope. In this manuscript we have shown implementations of SM-OCT for surface and retinal imaging. An important application for OCT is internal diagnostics using endoscopes[1,2]. A potential design for applying time-varying local phase changes in OCT endoscopes or needles for interstitial tissue imaging is provided above and shows the potential of SM-OCT for endoscopy applications, however, it does not take into consideration the likely challenging design of mechanics and electronics. Particularly, it may be difficult to miniaturize a motor with adequate rotational velocity for SM-OCT. This design for an SM-OCT endoscope is analogous to the system we implemented for this manuscript (Fig. 1b). SMF, single mode fiber; GRIN, gradient-index.

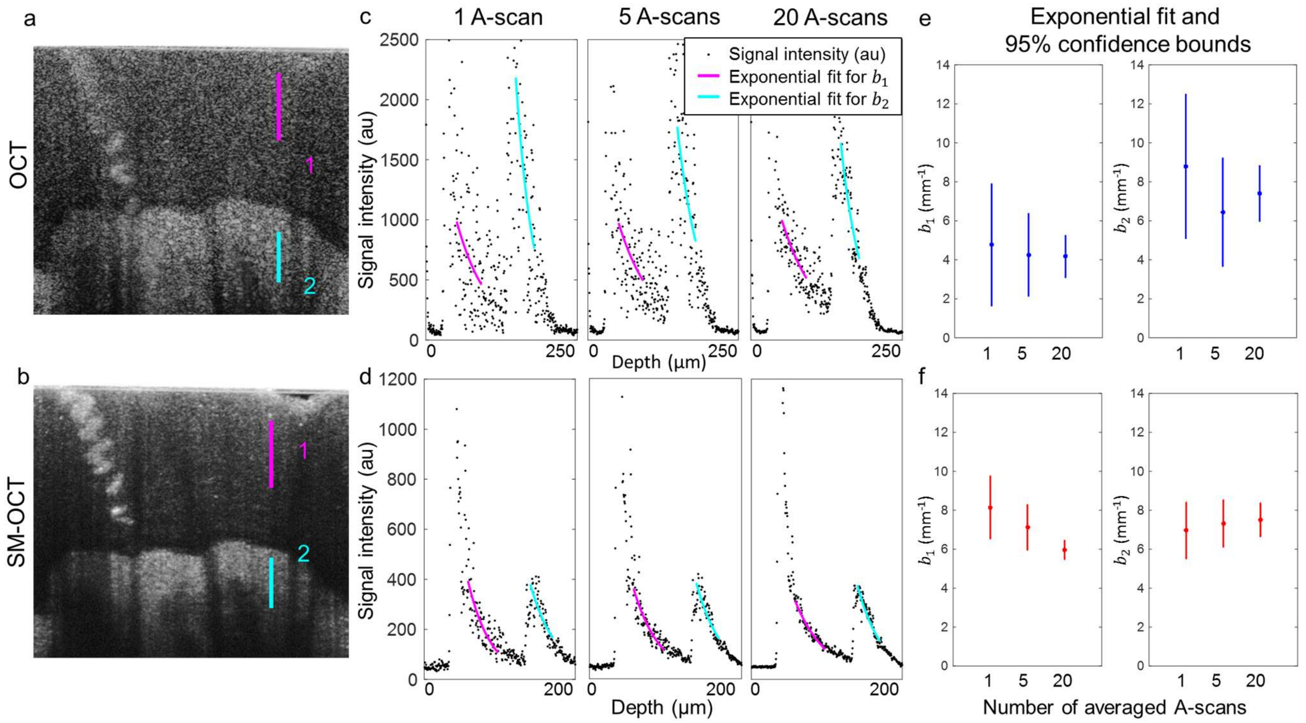

Supplementary Figure 23| SM-OCT enables a more accurate exponential fit which can be used for calculating the tissue attenuation coefficient. (**a,b**) OCT and SM-OCT B-scans of a fingertip showing a sweat duct. Magenta and cyan lines indicate the regions used for the calculation of the exponential fit parameters, b_1 and b_2, which are described by Supplementary Eq. 18. (**c,d**) Pixel values (in linear scale) and exponential fit. The calculation was performed for OCT and SM-OCT based on pixel values of 1 A-scan and based on intensity averaged values of 5 and 20 adjacent A-scans. (**e,f**) The calculated exponential coefficients, b_1 and b_2, with error bars representing the 95% confidence bounds. Tighter bounds indicate a more precise measurement. In SM-OCT the measurement of the exponential coefficient is more precise in all cases owing to reduced speckle noise. In order to achieve a higher precision with OCT, adjacent A-scans should be averaged, thereby reducing the spatial resolution of the measurement.

Supplementary Table 1. Incident power on the sample with conventional OCT and with the different diffusers.

	Mean (μW)	SD (μW)	Relative transmission	Power loss	Diffuser transmission	Diffuser power loss
OCT	814.75	4.24				
No diffuser	738.25	2.61	91%	9%		
3 μm lapped	574.85	1.83	71%	29%	78%	22%
2000 grit	593.55	1.9	73%	27%	80%	20%
1500 grit	555.7	2.05	68%	32%	75%	25%

Supplementary Table 2. Signal intensity with conventional OCT and with the different diffusers.

	mean signal intensity (au)	SD (au)	Signal loss
no diffuser	3.559	1.238	
3 µm lapped	2.285	0.812	36%
2000 grit	2.075	0.598	42%
1500 grit	1.769	0.486	50%

Supplementary Table 3. The effective lateral resolution of OCT and SM-OCT, calculated from the visibility of line separation in the resolution target, as shown in Supplementary Fig. 6.

	Smallest resolvable group (element) along vertical	Line pairs per mm	Effective FWHM (um)	Smallest resolvable group (element) along horizontal	Line pairs per mm	Effective FWHM (um)	PSF size increase
OCT	6 (6)	114	8.8	7 (3)	161	6.2	
SM-OCT, 2000	6 (6)	114	8.8	7(2)	144	6.9	6.18%
SM-OCT, 1500	6 (1)	64	15.6	6(6)	114	8.8	35.27%

Supplementary Table 4. The acquisition parameters for all images in this study.

Figure	Frame averaging	Pixel spacing x (µm)	Pixel spacing y (µm)	Diffuser used
2 a, c	30	2	2	-
2 b, d	30	2	2	1500
3 a	100	2	-	-
3 b	100	2	-	1500
4 b, d	100	2	-	-
4 f	5, 10, 20, 40, 100	2	-	-
4 c, e	100	2	-	1500
4 g	5, 10, 20, 40, 100	2	-	1500
5 a, c, e	100	2	-	-
5 b, d, f	100	2	-	1500
5 g	20	4	4	-
5 h	20	4	4	1500
6 a, c	66*	2	-	-
6 b, d	87*	2	-	1500
6 f, h	69*	2	-	-
6 g, i	81*	2	-	1500
7 a, b, c	20	4	4	-
7 d, e, f	100	2	-	2000
7 h	20	4	4	-
7 i	20	4	4	2000
Supp. 6 a, d	54	3	3	-
Supp. 6 b, e	54	3	3	2000
Supp. 6 c, f	54	3	3	1500
Supp. 8 c	30	2	2	-
Supp. 8 d	30	2	2	2000
Supp. 8 e	30	2	2	1500
Supp. 10	See caption	2	-	None/1500
Supp. 11	100	3	-	None/1500
Supp. 13 b	100	2	-	-
Supp. 13 c	100	2	-	1500
Supp. 14	See caption			
Supp. 15 a, c, d	100	2	-	-
Supp. 15 b	100	2	-	1500
Supp. 16	100	2	-	-
Supp. 18 a	100	2	-	-
Supp. 18 b	100 A-scan averages	2	-	-
Supp. 18 c	100 A-scan averages	2	-	1500
Supp. 19 a, 23 a	100	2	-	-
Supp. 19 b, 23 b	100	2	-	1500
Supp. 20	20	4	4	2000
Supp. 21 a, c	44	3	-	-
Supp. 21 b, d	44	3	-	3 µm
Supp. Movie 1-3	20	4	4	None/1500
Supp. Movie 4	20	4	4	None/ 2000

A blank entry for pixel spacing in the *y* axis refers to a 2D scan, as opposed to a 3D scan. A blank entry for the diffuser used refers to a conventional OCT scan, as opposed to a SM-OCT scan. *100 frames were acquired and several frames were removed from the average due to movement of the sample (see the Methods, Post processing section).

Supplementary Note 1: Simulation of speckle reduction with local-random phase variations

A Monte-Carlo simulation was implemented to simulate the reduction of speckle noise in turbid media using local-random phase variations. Supplementary Eq. 1 depicts an approximation of the signal intensity originating from a voxel inside a turbid sample.

$$I = \frac{1}{M}\sum_{m=1}^{M}\left|\sum_{n=1}^{N} a_n e^{i\varphi_n} e^{i\theta_{n,m}}\right| \qquad \text{(Eq. 1)}$$

I is the pixel value after averaging M scans obtained at different times and with different local phase shifts within the illuminating beam. N is the number of scatterers inside a voxel, and for each scatterer n within that voxel, a_n is its scattering amplitude (proportional to its amplitude reflection coefficient) and φ_n is the phase delay due to its axial location. $\theta_{n,m}$ is the local phase shift of the illumination beam, which changes in time in Speckle-Modulating OCT (SM-OCT), at the location of scatterer n.

Supplementary Fig. 1 shows that increasing the number of averages narrows the pixel value distribution, thereby reducing speckle noise. In the case of a random number of particles in each voxel, the pixel intensity variation persists with increased averaging owing to the actual variation of scatterers within each voxel. This underlying variation is why the distribution does not continue to narrow when increasing the number of averages from 100 to 1000.

The Monte-Carlo simulation considers 10,000 voxels. The number of particles in each voxel is either 30 or randomly chosen from a Poisson distribution with mean λ=30. The scattering amplitude is uniform across all particles and equal to 1. The phase contributed by each particle, φ_n, is a random variable with a uniform distribution between 0 and 2π. In OCT $\theta_{n,m}$ is constantly equal to zero; in SM-OCT it is a random variable with a uniform distribution between 0 and 2π.

Supplementary Note 2: Model and simulation of SM-OCT

Modeling and simulating the SM-OCT system, as shown in Fig. 1b, is beneficial for understanding and predicting the effect of different diffusers on image quality and speckle reduction. In this section, we present a simplified model for the SM-OCT sample arm based on Fourier optics (Supplementary Fig. 3a). This model was able to show that a fine-grit diffuser achieves slower changes in speckle compared to a rougher diffuser, a property of SM-OCT that we verified experimentally.

The electric field of the illuminating OCT beam, E_1, is approximated by a Gaussian spatial distribution, $\mathrm{PSF}_{\mathrm{L1}}$, defined by the first lens in the OCT sample arm, L1. The phase of the beam will be altered upon passing through the diffuser. The diffuser introduces a locally varying phase that results from the difference in the refractive index of glass and air, Δn, and the varying thickness of the ground glass, $d(x_{\mathrm{d}}+x, y_{\mathrm{d}}+y)$, with $(x_{\mathrm{d}}, y_{\mathrm{d}})$ representing the location of the center of the beam on the diffuser and (x, y) representing the position relative to the center of the OCT beam. An approximation of the local phase variation introduced by the diffuser is $\phi_{\mathrm{diffuser}}(x_{\mathrm{d}}+x, y_{\mathrm{d}}+y) = k\Delta n d(x_{\mathrm{d}}+x, y_{\mathrm{d}}+y)$, in which k is the average wave-number of the OCT illumination (note that a full description of the phase would require a vectorial electromagnetic simulation). The electric field immediately after passing through the diffuser is therefore[3]:

$$E_2(x_{\mathrm{d}}+x, y_{\mathrm{d}}+y) = E_1(x, y)\exp[ik\Delta n d(x_{\mathrm{d}}+x, y_{\mathrm{d}}+y)] \qquad \text{(Eq. 2)}$$

The OCT beam with this phase variation is then imaged from the diffuser plane onto the sample by two similar lenses, L2, in a 4f imaging configuration. These lenses smooth the beam's spatial features by a point-spread function, $\mathrm{PSF}_{\mathrm{4f}}$, due to the limited pupil size of the lenses and the finite size of the OCT beam. The resulting electric field on the sample is:

$$E_3(x_{\mathrm{d}}+x, y_{\mathrm{d}}+y) = E_2(x_{\mathrm{d}}+x, y_{\mathrm{d}}+y) * \mathrm{PSF}_{\mathrm{4f}}(x, y) \qquad \text{(Eq. 3)}$$

where $*$ is a convolution across the beam. The sample is simulated as many individual scatterers with certain sizes and random locations that reflect the incident electric field with an amplitude ratio of 1 and a random phase, $\phi_{\mathrm{sample}}(x, y)$, in the interval [0, 4π] (Supplementary Fig. 3b). This interval was chosen according to the axial resolution of the OCT (also referred to as the "coherence gating" of the OCT) which was 2 μm in our system. Therefore, the electric field immediately after the sample is:

$$E_4(x_{\mathrm{d}}+x, y_{\mathrm{d}}+y) = E_3(x_{\mathrm{d}}+x, y_{\mathrm{d}}+y)\exp\left[i\phi_{\mathrm{sample}}(x, y)\right] \qquad \text{(Eq. 4)}$$

The beam is now propagated back through the two L2 lenses and the diffuser:

$$E_5(x_{\mathrm{d}}+x, y_{\mathrm{d}}+y) = E_4(x_{\mathrm{d}}+x, y_{\mathrm{d}}+y) * \mathrm{PSF}_{\mathrm{4f}}(x, y) \qquad \text{(Eq. 5)}$$

$$E_6(x_{\mathrm{d}}+x, y_{\mathrm{d}}+y) = E_5(x_{\mathrm{d}}+x, y_{\mathrm{d}}+y)\exp[ik\Delta n d(x_{\mathrm{d}}+x, y_{\mathrm{d}}+y)] \qquad \text{(Eq. 6)}$$

The electric field E_6 then propagates through lens L1, where it interferes with a reference field E_{r}. In our simulation, we assumed that the reference field was identical to the initial field in the sample arm and that both were Gaussian beams that defined by $\mathrm{PSF}_{\mathrm{L1}}$. The phase of E_{r} was set to maximize the OCT signal intensity for each simulated sample in a setup with a smooth (zero-phase) diffuser. The OCT

signal intensity is calculated as the real component of the integration over the product of the sample and reference field[4,5]:

$$I_{\mathrm{OCT}}(x_{\mathrm{d}}, y_{\mathrm{d}}) \propto \mathrm{Re}\left\{\iint [E_6(x_{\mathrm{d}} + x, y_{\mathrm{d}} + y) * \mathrm{PSF}_{\mathrm{L1}}(x, y)] E_{\mathrm{r}}^*(x, y) \mathrm{d}x \mathrm{d}y\right\} \qquad \text{(Eq. 7)}$$

As the diffuser moves, the location of the beam on the diffuser, $(x_{\mathrm{d}}, y_{\mathrm{d}})$, changes, and if the local phase variations contributed by the diffuser are sufficient, the OCT signal may also vary.

In our simulation, $\mathrm{PSF}_{\mathrm{L1}}$ and $\mathrm{PSF}_{4\mathrm{f}}$ were approximated as Gaussian beams with a full-width at half-maximum (FWHM) of 8 µm and 4.2 µm, respectively, similar to the lenses used in our setup. The sample was simulated as 4,000 scatterers with size 0.8x0.8 μm^2. The wave-number, k, was 0.9 µm. The thickness of the diffusers was obtained from a white light interferometer measurement (Supplementary Fig. 4) and Δn was approximated as 0.5. The diffuser was moved in one dimension with increments of 0.25 µm and a range of 154 µm. We simulated a smooth diffuser, which contributes zero phase, a fine diffuser (2000 grit) and a rougher diffuser (1500 grit). The rougher diffuser is the one used in most of this study. The simulated OCT signal is presented in Supplementary Fig. 3d.

We calculated the normalized auto-covariance of the OCT signal in order to determine the amount of diffuser displacement that is needed in order to change the OCT signal, and therefore, change the speckle pattern. The normalized auto-covariance at displacement δ is calculated as:

$$\frac{\sum_{x_{\mathrm{d}}}(I_{\mathrm{OCT}}(x_{\mathrm{d}}, 0) - \overline{I_{\mathrm{OCT}}})(I_{\mathrm{OCT}}(x_{\mathrm{d}} + \delta, 0) - \overline{I_{\mathrm{OCT}}})}{\sum_{x_{\mathrm{d}}}(I_{\mathrm{OCT}}(x_{\mathrm{d}}, 0) - \overline{I_{\mathrm{OCT}}})^2} \qquad \text{(Eq. 8)}$$

in which $\overline{I_{\mathrm{OCT}}}$ is the average OCT signal over all the diffuser locations. Note that the denominator is simply the variance of the OCT signal. The simulation reveals that the OCT signal becomes decorrelated at a displacement of 7 µm and 15 µm for the rougher and finer diffusers, respectively (Supplementary Fig. 3e).

By imaging a single location in a phantom made of TiO_2 powder dispersed in PDMS with SM-OCT and a rotating diffuser, we were able to verify that the decorrelation of speckle indeed requires a larger diffuser displacement when using the finer diffuser compared to the rougher diffuser (Supplementary Fig. 3f-h). In the experiment, the decorrelation of the OCT signal occurs at smaller displacements compared to the simulation, by about a factor of 3. This discrepancy shows that the local phase variations in reality are larger compared to the simulation. A more accurate model of SM-OCT would require: an exact model for the interaction of the diffuser and the electric field, a more accurate optical simulation[4], and a simulation of spectral-domain OCT with coherence gating, confocal detection, and multi-layer scattering.

Supplementary Note 3: Measurement of gap in phantom

Images of the PDMS-TiO_2 phantom acquired with a bright field microscope (10x, NA=0.25), OCT, and SM-OCT were registered and segmented to detect the gap in the phantom (Fig. 2). Identical cross-section planes (*en face*) were chosen from the 3D scans acquired with OCT and SM-OCT. Because these scans were taken consecutively, only minor translation adjustments were required in order to register the cross-sections. The microscope image was scaled using the known pixel size ratio between the OCT and microscope, and this image was registered to the OCT and SM-OCT scans manually with translation and rotation (Supplementary Fig. 8d). The segmentation threshold of the OCT and SM-OCT scans was chosen carefully so that a maximal number of pixels will be considered inside the gap, but without large holes inside the phantom. Small holes were filled with the morphological close operator with a structure element of size 2 (MATLAB function imclose), which is equivalent to 4 µm. The threshold was chosen as the lowest value in which the phantom had no holes inside it. After finding the segmentation threshold, the original image was segmented, without morphological closing (Supplementary Fig. 8a-c). In order to exclude holes in the measurement of the gap while retaining its shape and size, holes were filled using the imfill function. Segmentation of the microscope image was performed using Otsu's method (graythresh function). The gap was measured by counting the number of pixels belonging to the gap across the x axis of the phantom (Supplementary Fig. 9).

Supplementary Note 4: Analysis of speckle and sample statistics

OCT speckle noise follows a Rayleigh distribution (Supplementary Eq. 9). SM-OCT reduces speckle and shifts the pixel value statistics from a Rayleigh distribution toward the expected distribution of scatterers in a sample: a Poisson distribution[6], in which each scattering event within a voxel contributes a signal value that is equal to the backscattering of an individual nanoparticle (Supplementary Eq. 10).

The expressions for the two distributions are:

$$p_{\mathrm{rayleigh}}(I) = \frac{\pi I}{2\langle I\rangle^2}\exp\left[-\frac{\pi I^2}{4\langle I\rangle^2}\right] \qquad \text{(Eq. 9)}$$

$$p_{\mathrm{poisson}}(k) = \frac{\lambda_p^k e^{-\lambda_p}}{k!}, \qquad p_{\mathrm{poisson}}(I) = I_p \times p_{\mathrm{poisson}}(k) \qquad \text{(Eq. 10)}$$

in which I is the pixel value, $\langle I\rangle$ the average pixel intensity, and I_p is the intensity of the backscatter from a single nanoparticle. k is the number of particles in a voxel, each of which we assume contributes equally to the OCT signal and λ_p is the average number of particles in a voxel.

Statistical analysis of the pixel values with OCT and SM-OCT (Fig. 3c-d) was performed by analyzing images of an agarose-GNS phantom. The 2D ROI was chosen at a similar depth inside the phantom, at a narrow region around the image plane. The statistical analysis was performed on the pixel values on a linear scale.

Supplementary Note 5: Characterization and reduction of illumination variability

The diffuser introduces a variability in the illumination intensity of the sample (Supplementary Fig. 10). This variability is sometimes referred to as "speckle illumination,"[7] and its effects can be reduced by averaging several scans acquired with the diffuser at different locations, which is also needed to remove speckle noise. In addition to reducing speckle noise, the illumination of the sample through the diffuser reduces unwanted diffraction artifacts from optical elements and dust particles in the beam path[7–10]. It is interesting to note that by introducing an external speckle illumination noise, we are able to remove the speckle noise which originates from within the sample. The variability of the external speckle illumination, implemented by temporal randomization, enables SM-OCT to create images with significantly improved quality.

In order to characterize the illumination variability and its reduction by averaging, we imaged a glass slide with OCT and SM-OCT (Supplementary Fig. 11a-c). In the OCT image, there are visible diffraction artifacts, manifested as circular Airy patterns that are likely due to dust particles. The SM-OCT images were acquired by averaging 100 frames with a static and moving diffuser. In the scan acquired with a static diffuser, the illumination variability is clearly visible. When averaging frames that were acquired as the diffuser was moving, the illumination variability reduces significantly, although some variability still exists. The histograms of the normalized pixel intensities (Supplementary Fig. 11d) show that the distribution of the SM-OCT signal changes from a speckle (Rayleigh) distribution to a much narrower distribution as the diffuser moves. Furthermore, the distribution of the pixel intensities in the SM-OCT scan with a moving diffuser is narrower than the distribution of the pixel intensities in the OCT scan, owing to the reduction of diffraction artifacts.

Averaging frames acquired through different locations of the diffuser reduces the effect of illumination variability, however, residual artifacts may remain if the SM-OCT images are acquired with a small number of averages, which may be needed in order to image moving living samples. This effect is particularly visible in A-scan averaging based SM-OCT, compared to frame (B-scan) averaging, because in A-scan based SM-OCT, the diffuser moves a shorter distance between each averaged A-scan. Because the illumination variability creates darker and brighter A-scans throughout the depth of the sample, we are able to reduce the artifacts of residual speckle illumination in post processing. If there is remaining variability, we can compensate for it by amplifying each A-scan adaptively, by assuming that the average signal across the sample does not change very quickly. The algorithm used for postprocessing is described below:

$$\bar{I}(x_n) = \frac{1}{M}\sum_{m=1}^{M} I^{\text{initial}}(x_n, z_m) \qquad \text{(Eq. 11)}$$

$$I^{\text{final}}(x_n, z_m) = I^{\text{initial}}(x_n, z_m) \times \frac{LPF\{\bar{I}(x_n)\}}{\bar{I}(x_n)} \qquad \text{(Eq. 12)}$$

x_n and z_m denote the discretized locations in the images along the lateral and axial dimensions, respectively. $I^{\text{initial}}(x_n, z_m)$ is the initial signal, in linear scale, after averaging A-scans and/or frames,

$\bar{I}(x_n)$ is the average signal along the depth of the image or a region inside the sample and $I^{\text{final}}(x_n, z_m)$ is the post-processed image. The LPF operator represents a low-pass filter, that can be implemented as a convolution with a Gaussian kernel or a median filter. This algorithm can be easily extended to a 3D volume by low-pass filtering the intensity of the 2D projection and by applying the adaptive gain to each A-scan in the volume.

In Supplementary Fig. 12 we compare SM-OCT scans of a PDMS + TiO_2 phantom with 20 A-scan averages, before and after compensation, to OCT and SM-OCT scans with 100 frame averages. The residual vertical lines (Supplementary Fig. 12d) are not visible after postprocessing (Supplementary Fig. 12e) and the TiO_2 aggregates can be observed. Low pass filtering was implemented by a median filter with a 50 pixel window.

Note that this post-processing method was not applied to any of the images in the manuscript. We found that algorithmic removal of the signal variation due to speckle illumination was only needed when performing fewer than 20 A-scan averages.

Supplementary Note 6: Analysis of speckle contrast reduction

To further validate that SM-OCT removes speckle noise, we compared experimental data to the theoretical decrease of speckle contrast, proportional to $1/\sqrt{M}$, where M is the number of compounded images with uncorrelated speckle noise. Including the inherent signal variation of the sample, the decrease in normalized standard deviation (SD), C, can be described by:

$$C = \frac{\sigma}{\langle I \rangle} \quad \text{(Eq. 13)}$$

$$C^2 \langle I \rangle^2 = \sigma^2 = \sigma_0^2 + \sigma_{\text{speckle}}^2 \quad \text{(Eq. 14)}$$

$$\sigma_{\text{speckle}}^2 = \frac{\sigma_{\text{speckle},0}^2}{M} \quad \text{(Eq. 15)}$$

Here, σ is the measured SD in a region of interest (ROI), and $\langle I \rangle$ the average pixel intensity in the same ROI. σ^2 is the measured variance in pixel values, σ_0^2 is the intrinsic variation in signal (in this case, caused by the variation in number of particles in a voxel), $\sigma_{\text{speckle}}^2$ is the variance of the speckle noise, which decreases by a factor of M during compounding, and $\sigma_{\text{speckle},0}^2$ is the variance of the speckle noise without any compounding. The values for σ_0^2 and $\sigma_{\text{speckle}}^2$ were found using a non-linear least-squares fit to Supplementary Eq. 14.

Since the normalized SD is composed of both speckle noise and the intrinsic random distribution of particles in the phantom (represented by σ_0), we define the normalized speckle (Supplementary Eq. 16), which decreases by a factor of $\sqrt{M}$ theoretically (predicted by Supplementary Eq. 15) and experimentally (Fig. 3f).

$$\text{Normalized speckle} = \sqrt{\frac{\sigma^2 - \sigma_0^2}{\sigma_{\text{speckle},0}^2}} = \frac{1}{\sqrt{M}} \quad \text{(Eq. 16)}$$

Supplementary Note 7: Comparison to existing methods of speckle reduction

SM-OCT was compared to common alternative speckle reduction techniques. Spatial compounding, one of the most common methods for reducing speckle, was implemented in two ways. The first method applied 3D Gaussian smoothing on a volume acquired with conventional OCT (Supplementary Fig. 14c-d). Smoothing was performed on the linear-scale image (after resampling) using Matlab's smooth3 function with a Gaussian kernel in a square window with a size of 11 pixels. The standard deviation of the Gaussian was 0.95 and 1.25. The second method attempted to reduce speckle by averaging adjacent frames along the *y* axis (Supplementary Fig. 14e-h). Averaging was performed on the linear-scale images over 4, 7, 9, and 13 frames, respectively (spanning 12, 24, 32, and 48 μm). These images show that although spatial compounding reduces speckle noise, it does not reveal structure as well as SM-OCT.

Polarization diversity is expected to provide minimal speckle noise reduction due to the limited number of possible uncorrelated speckle patterns[11,12]. Frequency compounding requires reconstructing the OCT image from several sub-bands. Compounding a large number of non-overlapping bands would reduce the axial resolution considerably. Therefore, these two methods were not examined. Angular compounding often produces favorable results. However, it is limited in its capability to reduce speckle due to the limited number of obtainable uncorrelated speckle patterns as a result of the overlap of the beams illuminating the sample at different angles[13–16]. Desjardins et al[13] have demonstrated speckle noise removal that is equivalent to compounding 30 uncorrelated speckle patterns, which provides a substantial improvement in image quality and can theoretically be extended if applied on the solid angle instead of a single plane. However, such removal is still limited in the number of uncorrelated speckle patterns it can produce.

Digital removal of speckle noise is also widely used. We compared SM-OCT images to OCT images that were post-processed with one of three digital methods with several different parameters. The filters were applied on a logarithmic scale OCT image[17]. Adaptive Wiener filtering was implemented with the Matlab function wiener2, using neighborhoods of sizes 5x5 and 7x7 pixels (Supplementary Fig. 15c-d). Hybrid median filter (HMF) was implemented[18] using neighborhoods of sizes 5x5, 7x7 and 9x9 pixels (Supplementary Fig. 16a-c). Symmetric nearest-neighbor filter (SNN) was implemented[19] using neighborhoods of sizes 5x5, 7x7 and 9x9 pixels (Supplementary Fig. 16d-f). These examples show that filters of a small size do not reduce speckle noise sufficiently, whereas larger filters create artifacts and compromise some of the information in the image.

Supplementary Note 8: SM-OCT with A-scan averaging

To enable more robust SM-OCT imaging of moving samples, we implemented our approach using A-scan averages instead of frame (B-scan) averages. For this purpose, the diffuser was moved rapidly and continuously using a rotating mount, providing uncorrelated speckle patterns in each A-scan (Supplementary Fig. 17).

We analyzed the correlation between speckle patterns in this acquisition scheme by imaging a single location in a phantom made of TiO_2 powder dispersed in PDMS. First, we imaged the phantom with the diffuser not moving. This measurement resulted in a static speckle pattern that is constant throughout the A-scans (Supplementary Fig. 17a). Next, we imaged the same location with the diffuser rotating at a velocity of 9 mm s^{-1} at the scanning location. At this velocity and an A-scan rate of 20 kHz, we obtain a translation of 0.45 µm per A-scan. These conditions resulted in a quickly-varying speckle pattern and rapid decorrelation (Supplementary Fig. 17b).

The speed of variation of the SM-OCT signal, which is equivalent to the change in speckle, can be measured by calculating the normalized auto-covariance using Supplementary Eq. 8 (Supplementary Fig. 17c). This measurement shows that the normalized auto-covariance decreases very rapidly, to a value of 0.2, within one A-scan, confirming the visibly rapid changes in the speckle pattern following sub-micron displacements of the diffuser.

To further analyze the correlation between speckle patterns in different A-scans we calculated the normalized cross-covariance as follows:

$$\mathrm{CovMat}_{ij} = \frac{1}{Z}\sum_{z=1}^{Z} \frac{(I_{i,z}-\bar{I}_i)(I_{j,z}-\bar{I}_j)}{\sigma_{I_i}\sigma_{I_j}} \qquad \text{(Eq. 17)}$$

In which CovMat_{ij} denotes the *i,j* values of the cross-covariance matrix, $I_{i,z}$ is the signal intensity of the ith A-scan in depth z in a region of interest (inside the phantom) that has Z rows. $\bar{I}_i$ and σ_{I_i} are the average and standard deviation of the ith A-scan along the depth dimension, respectively. The scan with the static diffuser produced a high cross-covariance between all A-scans (Supplementary Fig. 17d). However, when the diffuser was moving, this value rapidly decreased, even between adjacent A-scans (Supplementary Fig. 17e). The cross-covariance value is affected by the structure of the phantom, in addition to the speckle noise. To obtain a value for the cross-covariance of speckle patterns and remove the influence of the signal of the phantom, we also calculated the cross-covariance after subtracting the average signal across A-scans. This calculation yielded cross-covariance values very close to zero outside of the diagonal, showing that speckle patterns of different A-scans were decorrelated when the diffuser was moving (Supplementary Fig. 17f).

We used SM-OCT with A-scan averaging to image the cornea of a mouse *in vivo* (Supplementary Fig. 18).

Supplementary Note 9: Measurement of the attenuation coefficient

Studies have shown that using OCT to measure the local attenuation coefficient in tissue can provide diagnostic information, such as the detection of tumor margins[20]. The attenuation coefficient of a sample may be calculated by fitting the OCT or SM-OCT signal intensity to a function which includes the effects of the Beer-Lambert law (an exponential function), the confocal function, OCT roll-off and multiple scattering[21,22].

We expect that SM-OCT would enable a more precise fit owing to the removal of speckle noise. In order to compare the precision of the fit between OCT and SM-OCT, we performed an exponential fit to the signal intensity. This simplified model assumes that tissue attenuation, governed by the Beer-Lambert law, is the most dominant factor in the decrease of signal intensity as a function of depth. We compared the precision of the fit between OCT and SM-OCT on an image of a fingertip (Supplementary Fig. 23). The precision of the fit can be determined by the 95% confidence bounds.

The Beer-Lambert law is given by the following expression[22]:

$$i(z) \propto \sqrt{\exp(-2\mu z)} = \exp(-bz) \qquad \text{(Eq. 18)}$$

in which $i(z)$ is the depth dependent OCT or SM-OCT signal intensity and μ is the attenuation coefficient. b is the exponential coefficient that we are attempting to find by fitting the signal to an exponential function.

The measurement of the exponential coefficient was more precise in SM-OCT images compared to OCT images in all cases owing to reduced speckle noise. In order to achieve a higher precision with OCT, adjacent A-scans should be averaged, thereby reducing the spatial resolution of the measurement. We showed the increase in precision following A-scan averaging by averaging the intensity of 5 and 20 adjacent A-scans.

Supplementary Discussion

One may intuit that the addition of the diffuser would degrade the lateral and axial resolution and significantly reduce the signal-to-noise ratio of SM-OCT. Indeed, the addition of random local phase shifts introduces aberrations to the illuminating wavefront, which will inevitably broaden the waist of the focused beam and reduce the lateral resolution. However, as described below, the measurements presented in this study show that the compromise in resolution in SM-OCT is insignificant. Moreover, the resolution of SM-OCT images of turbid media is consistently superior to that of OCT images owing to the removal of speckle noise.

The axial resolution of the system is preserved owing to the averaging of the thickness variations of the diffuser over time. This averaging is why features as small as 2 μm are resolved along the vertical direction (such as the dark line in Fig. 5f).

The lateral resolution of SM-OCT was measured using a resolution test target. The results show an increase of 35.3 % and 6.2 % in the size of the point spread function (PSF) with the 1500 grit and 2000 grit diffusers, respectively (Supplementary Fig. 6, Supplementary Table 3). We attribute the increase in size of the PSF to the aberration of the wavefront when it passes through the diffuser. The diffuser creates a variable phase across the wavefront that translates into a somewhat aberrated and larger PSF after the 4f imaging system owing to the limited pupil size of these lenses. We believe that the difference between the two diffusers was caused by the increased roughness of the 1500 grit diffuser, which resulted in deflection of light and local loss of signal. The local signal variations were not completely removed by the 54 averages used to create these images. It is conceivable that the diffuser topography can be optimized to reduce the size of the PSF while also creating the phase variability required to reduce speckle by creating non-correlated speckle patterns.

While the lateral resolution of SM-OCT was degraded compared to OCT when measured on a glass test chart, the visibility of closely-spaced scattering objects of SM-OCT was superior when imaging turbid samples, which are ordinarily dominated by speckle. We have measured a 250% increase in the *effective* resolution measured by SM-OCT compared to conventional OCT (Fig. 2). The achievable increase in visibility owing to SM-OCT may be much higher in features with lower contrast, which are usually completely hidden by speckle noise in conventional OCT.

The optical power and signal intensity were measured for all three diffusers and compared to conventional OCT (Supplementary Fig. 5, Supplementary Table 1,2). In the HR-OCT system, the power on the sample decreases by 9% due to the 4f imaging system, and an additional reduction of 22%, 20%, and 25% is due to the 3μm lapped, 2000 grit and 1500 grit diffusers, respectively. The OCT and SM-OCT signals were measured from a PDMS-TiO_2 phantom and yielded an average signal loss of 36%, 42%, and 50% with the three diffusers compared to OCT. We attribute the reduction of optical power, and subsequent reduction of OCT signal, to the roughness (rapid variations of thickness) of the diffuser. High gradients in the thickness of the diffuser result in local deflection of light which is then unable to contribute to the OCT signal. The local loss of light is apparent in OCT images as darker A-scans

(Supplementary Fig. 10). While the current implementation of SM-OCT suffers from a lower signal-to-noise ratio, the technique is still able to produce images with better visibility of fine features compared to OCT. Note that the noise in pixels within the tissue was much higher than that within pixels above the tissue (Fig. 5a-b, for example), indicating that speckle noise was more significant than photon shot noise and sensor readout noise in our measurement (after several averages). In most applications, the reduction in signal intensity can be compensated by increasing the incident power on the sample.

In the retinal OCT system described herein, in addition to not being able to increase the power of the light source (due to practical limitations of the instrument), we did not have access to the raw scan data. Thus, we were unable to optimize the acquisition and post-processing algorithms in order to produce higher quality images with the iFusion. To circumvent these limitations, we created a finer grit diffuser by lapping a 1500 grit diffuser with 3 μm particles. The finer diffuser produced images with a higher signal-to-noise ratio but possibly at the expense of effective speckle noise reduction. Although speckle may still be present in the retina images, the retinal layers are better defined in SM-OCT when compared to conventional OCT (Supplementary Fig. 21).

Supplementary References

1. Tearney, G. J. *et al.* In vivo endoscopic optical biopsy with optical coherence tomography. *Science* **276,** 2037–2039 (1997).
2. Yicong Wu, Y. *et al.* Robust High-Resolution Fine OCT Needle for Side-Viewing Interstitial Tissue Imaging. *IEEE J. Sel. Top. Quantum Electron.* **16,** 863–869 (2010).
3. Goodman, J. W. *Speckle Phenomena in Optics: Theory and Applications*. (Roberts and Company Publishers, 2007).
4. Thrane, L., Yura, H. T. & Andersen, P. E. Analysis of optical coherence tomography systems based on the extended Huygens–Fresnel principle. *J. Opt. Soc. Am. A* **17,** 484–490 (2000).
5. Schmitt, J. M. & Knüttel, A. Model of optical coherence tomography of heterogeneous tissue. *J. Opt. Soc. Am. A* **14,** 1231–1242 (1997).
6. Benedek, G. B. & Villars, F. M. H. in *Physics With Illustrative Examples From Medicine and Biology: Statistical Physics* 288–290 (Springer Science & Business Media, 2000).
7. Park, Y. *et al.* Speckle-field digital holographic microscopy. *Opt. Express* **17,** 12285–12292 (2009).
8. Dubois, F., Requena, M. L. N., Minetti, C., Monnom, O. & Istasse, E. Partial spatial coherence effects in digital holographic microscopy with a laser source. *Appl. Opt.* **43,** 1131–1139 (2004).
9. Somekh, M. G., See, C. W. & Goh, J. Wide field amplitude and phase confocal microscope with speckle illumination. *Opt. Commun.* **174,** 75–80 (2000).
10. Pitter, M. C., See, C. W. & Somekh, M. G. Full-field heterodyne interference microscope with spatially incoherent illumination. *Opt. Lett.* **29,** 1200–1202 (2004).
11. Schmitt, J. M., Xiang, S. H. & Yung, K. M. Speckle in optical coherence tomography. *J. Biomed. Opt.* **4,** 95–105 (1999).
12. Storen, T., Royset, A., Giskeodegard, N. H., Pedersen, H. M. & Lindmo, T. Comparison of speckle reduction using polarization diversity and frequency compounding in optical coherence tomography. Proc. SPIE **5316,** 196–204 (2004).
13. Desjardins, A. E., Vakoc, B. J., Tearney, G. J. & Bouma, B. E. Speckle Reduction in OCT using Massively-Parallel Detection and Frequency-Domain Ranging. *Opt. Express* **14,** 4736–4745 (2006).
14. Desjardins, A. E. *et al.* Angle-resolved Optical Coherence Tomography with sequential angular selectivity for speckle reduction. *Opt. Express* **15,** 6200–6209 (2007).
15. Iftimia, N., Bouma, B. E. & Tearney, G. J. Speckle reduction in optical coherence tomography by 'path length encoded' angular compounding. *J. Biomed. Opt.* **8,** 260–263 (2003).
16. Hughes, M., Spring, M. & Podoleanu, A. Speckle noise reduction in optical coherence tomography of paint layers. *Appl. Opt.* **49,** 99–107 (2010).
17. Ozcan, A., Bilenca, A., Desjardins, A. E., Bouma, B. E. & Tearney, G. J. Speckle reduction in optical coherence tomography images using digital filtering. *J. Opt. Soc. Am. A.* **24,** 1901–1910 (2007).
18. Garcia, D. Hybrid median filtering. *MATLAB Central File Exchange* (2009).

19. Barnes, A. Symmetric nearest neighbor filter. *MATLAB Central File Exchange* (2005).

20. Kut, C. *et al.* Detection of human brain cancer infiltration ex vivo and in vivo using quantitative optical coherence tomography. *Sci. Transl. Med.* **7,** 292ra100 (2015).

21. Smith, G. T. *et al.* Automated, Depth-Resolved Estimation of the Attenuation Coefficient From Optical Coherence Tomography Data. *IEEE Trans. Med. Imaging* **34,** 2592–2602 (2015).

22. Faber, D. J., van der Meer, F. J., Aalders, M. C. G. & van Leeuwen, T. G. Quantitative measurement of attenuation coefficients of weakly scattering media using optical coherence tomography. *Opt. Express* **12,** 4353–4365 (2004).
